# Fine-scale spatial organisation of deep-sea sea pens in a NE atlantic submarine canyon conservation area

**DOI:** 10.1038/s41598-025-13327-2

**Published:** 2025-08-11

**Authors:** Irene Susini, Loïc Van Audenhaege, David M. Price, Tabitha R. R. Pearman, Emily G. Mitchell, Veerle A. I. Huvenne

**Affiliations:** 1 Ocean and Earth Science, University of Southampton Waterfront Campus, Southampton, SO14 3ZH UK; 2 School of Biological and Marine Sciences, University of Plymouth, Drake Circus, Plymouth, PL4 8AA UK; 3 National Oceanography Centre, European Way, Southampton, SO14 3ZH UK; 4 Institut de Ciències del Mar, Consejo Superior de Investigaciones Científicas (ICM-CSIC), Passeig Marítim de la Barceloneta, 37-49, Barcelona, 08003 Spain; 5 Institute of Marine Sciences - Okeanos, University of the Azores, Horta, Portugal; 6 Department of Zoology, University Museum of Zoology, University of Cambridge, Cambridge, UK

**Keywords:** Deep-sea sea pen, Pennatuloidea, Spatial point pattern analysis, Benthic habitat, Vulnerable marine ecosystem, Submarine canyon, Ecology, Community ecology

## Abstract

Sea pens are colonial octocorals inhabiting mostly muddy and sandy soft sediments, globally. Despite their cosmopolitan distribution, evidence of high functional value, and susceptibility to external stressors, sea pens remain poorly investigated relative to other coral species. Here, a quantitative analysis of the fine-scale spatial patterns of deep-sea sea pens (Octocorallia: Pennatuloidea) within a submarine canyon conservation area in English waters is conducted using Remotely Operated Vehicle (ROV) seabed images, three-dimensional photogrammetry models, and Spatial Point Pattern Analysis (SPPA). The density distribution of sea pens at the canyon flank scale (100s of metres) is also reported for context. At the canyon flank scale, sea pen density decreases with increasing depth, with colonies virtually absent from waters deeper than ca. 1100 m. At the fine scale, sea pens exhibit a tendency to cluster relative to sea pens and tube-dwelling anemones (Cerianthidae sp.), suggesting mutual habitat associations. Conversely, overdispersion best characterises the nature of sea pen distribution relative to stalked sponges (*Hyalonema* sp.), suggesting occurrence of competition or, possibly, allelopathy. This study demonstrates that high-resolution spatial analysis techniques can be employed to disentangle biotic interactions among a set of taxa, revealing key processes and structures underlying the ecology of benthic fauna.

**Supplementary Information:**

The online version contains supplementary material available at 10.1038/s41598-025-13327-2.

## Introduction

The marine environment is increasingly subjected to pervasive anthropogenic pressures, with ca. 59% of global waters experiencing intensifying cumulative impacts^1^. The deep ocean further faces disturbances of natural origin—e.g., variations in phytodetritus, circulation, and sedimentary regimes^2–4^—driving patterns in deep seascapes^5^. Vulnerable Marine Ecosystems (VMEs) are key components of the deep seascape which are “physically or functionally fragile”^6^ and “easily disturbed and very slow to recover, or (which) may never recover”^6^. VMEs often include habitat-forming organisms that create three-dimensional underwater structures^7–9^ which are susceptible to disturbance owing to the intrinsic vulnerability of their constituent parts^6^. Taxa such as scleractinian corals, sea pens, and sponges are considered indicators of VME presence in particular regions of the ocean, with occurrence of VME indicator taxa implemented routinely as a proxy for VME detection^10–12^. VMEs are further identifiable through habitat types formed by indicator taxa^13^, including Cold-Water Coral (CWC) reefs, sea pen fields, and deep-sea sponge aggregations. Not all VMEs, however, attract equal scientific interest, with sea pens remaining relatively poorly investigated compared to other VME indicator taxa, e.g., scleractinian corals^14^.

Sea pens (Octocorallia: Pennatuloidea) are colonial octocorals primarily inhabiting muddy or sandy soft sediments, globally^15^. Often occurring in dense, erect aggregations known as ‘sea pen fields’^16^, sea pens afford structural complexity in otherwise featureless areas of the seafloor, providing refugia^17,18^, attachment substrata^19,20^, and feeding opportunities for associated fauna^21,22^. Sea pen occurrence also likely alters water current flows at the seabed–water interface, retaining nutrients and entraining plankton near the sediment^23–25^. However, such insights are derived from limited observations, restricted to few species and/or geographical regions. Considering the cosmopolitan distribution of sea pens^26^, the full extent of their contribution to deep-sea biodiversity has yet to be elucidated, with crucial elements such as patch size and faunal associations remaining poorly understood. To ensure the health of VME habitats, their ecological dynamics and distribution patterns need to be understood more thoroughly^27^, through the generation of accurate, quantitative, and spatially explicit information at scales relevant to conservation objectives^28^.

Spatial faunal patterns act as ‘ecological archives’ of the underlying processes, structure, and function^29,30^ of benthic communities, including those formed by VME indicator taxa. Spatial Point Pattern Analysis (SPPA), a spatial analysis technique, enables the linkage of spatial patterns with the most likely underlying drivers^31^ of the ecological dynamics of benthic communities. SPPA can identify random, clustered, or overdispersed^32^ spatial patterns, which often change across spatial scales^29^. Clustering may reflect fragmentation and dispersal limitation in live scleractinian corals^33^ or habitat associations within or between taxa^34^, whilst non-random aggregation of dead glass sponge specimens has been linked to density-dependent mortality^35^. Investigations into the spatial distribution of benthic taxa, therefore, afford insights into the demographic strategies contributing to population dynamics^33^, information critical to predict the likely response of a population to anthropogenic pressures^36^ and assess long-term population viability^33^. Intra- and interspecific interactions are further key drivers of distribution patterns, community structure and functioning, and evolutionary changes^37^. Biotic interactions also affect the realised niche of species^38^, knowledge of which is essential for the protection of sensitive habitats^39^. SPPA is a particularly powerful tool for determining the ecological underpinnings of the point patterns of benthic taxa, as it is able to capture shifts in interspecific interactions and associations over distances of a few metres^35^. SPPA of discrete point data has long been applied to terrestrial systems, e.g., ^40^. However, in marine settings, particularly in the deep sea, the quantification of spatial faunal patterns remains challenging due to the technical difficulties inherent to the collection of positionally accurate biological data^41^.

The application of three-dimensional (3D) photogrammetry is particularly revolutionary for the characterisation of spatial faunal patterns in the deep sea, allowing for accurate registration of organisms’ relative positions^35,42,43^. Three-dimensional reconstructions achieved through the acquisition of consecutive, overlapping photographs from a single moving camera—a technique referred to as Structure-from-Motion (SfM)^44^—enable fine-scale assessments of benthic habitat morphology at the centimetric scale, and have been used in the deep sea to quantify fine-scale habitat complexity and describe spatial patterns of CWC reefs^35,41,45,46^; however, they have yet to be applied to sedimentary habitats such as sea pen fields.

This study aims to investigate the fine-scale spatial patterns of sea pen (Pennatuloidea spp.) assemblages with respect to other sea pens and co-occurring taxa (Cerianthidae sp. and *Hyalonema* sp.) within the Dangaard Canyon (NE Atlantic)—an area that forms part of The Canyons Marine Conservation Zone (MCZ), the only designated deep-sea MCZ in English waters. VME indicator taxa are known to attain large abundance in submarine canyons, the latter occupying 11.2% of global continental and island margins^47^ and acting as biodiversity hotspots owing to their complex topographies and hydrodynamic regimes^48,49^. The wider Whittard Canyon system, which encompasses the Dangaard Canyon, has been shown to experience faunal distribution variations over fine spatial scales^4,50^, rendering it a pertinent setting to investigate the ecological dynamics and spatial patterns of poorly understood species.

## Results

**Fig. 1:**
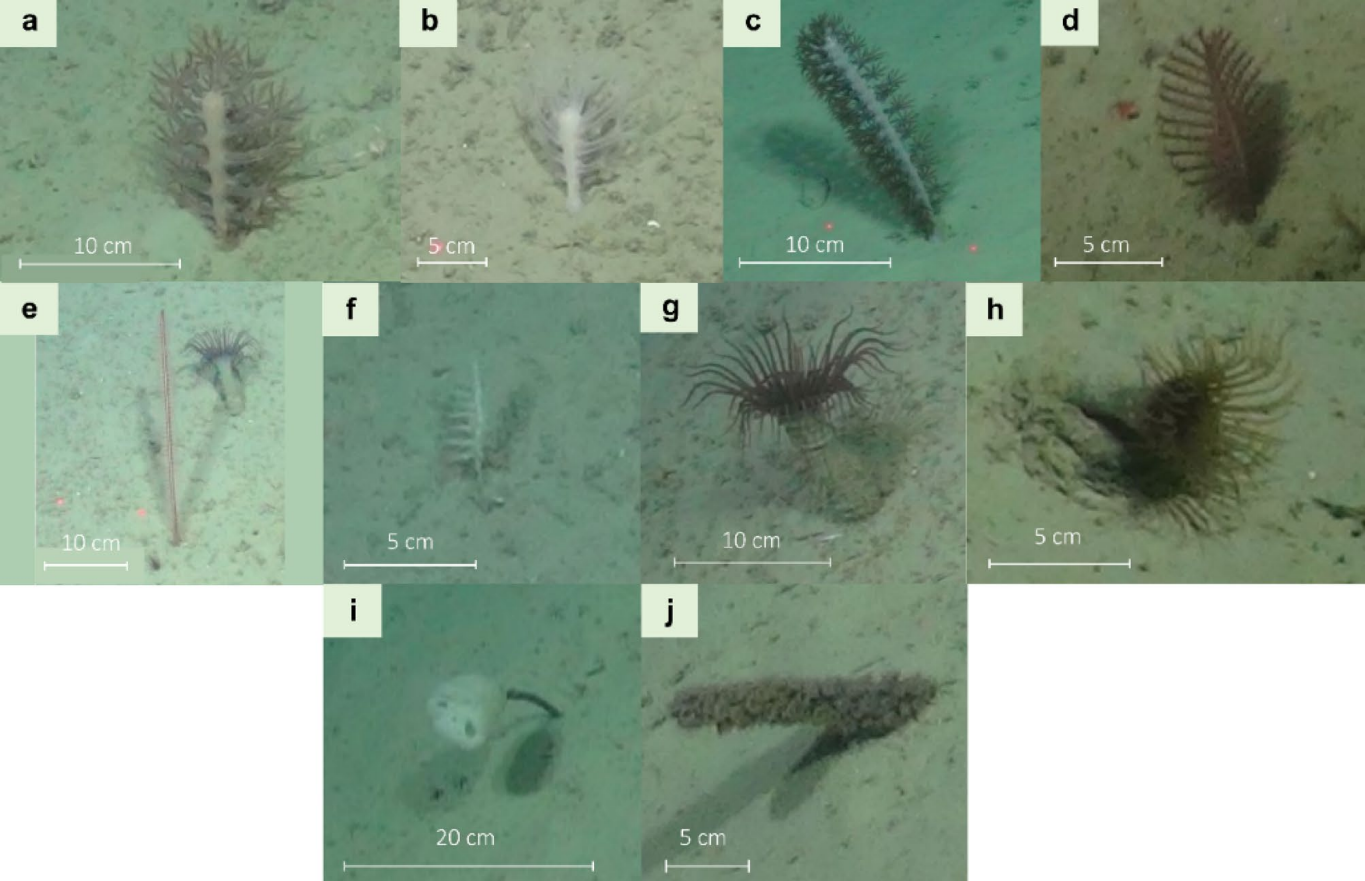
Morphospecies subject to investigation. (**a**) *Kophobelemnon* msp1; (**b**) *Kophobelemnon* msp1 (white); (**c**) *Kophobelemnon* msp2; (**d**) *Pennatula* sp.; (**e**) *Protoptilum* sp. (front) and Cerianthidae sp. (background); (**f**) *Virgularia* sp.; (**g**) Cerianthidae sp. (purple); (**h**) Cerianthidae sp. (brown); (**i**) *Hyalonema* sp. and (**j**) its stalk inhabited by Zoantharia msp7. Morphospecies classification was based on the SMarTaR-ID image repository^51^.

At the canyon flank scale, where analysis was based on Remotely Operated Vehicle (ROV) stills not included in the spatial analyses, a total of 104 images (442.5 m^2^) from the upper bathyal zone (686–1328 m) on the northern flank of the Dangaard Canyon were annotated across 13 consecutive 50 m depth bins (**Table ****S1**). A total of 296 specimens were identified as Pennatuloidea spp., co-occurring with one of two ‘dominant’ taxa: Cerianthidae sp. (tube-dwelling anemones) or *Hyalonema* sp. (stalked sponges; Fig. 1). A decrease in Pennatuloidea spp. density was documented with depth (**Table S2**).

At the fine scale, where analysis was based on 10 m subsections from raw ROV footage, a total of 10 reconstructed transects (371.7 m^2^) from the upper bathyal zone—between 671 ± 3.5 m (SD) and 1000 ± 5.7 m (SD)—contained enough specimens (*n* > 30^31^) to perform univariate and bivariate SPPA. A total of 420 Pennatuloidea spp. colonies were annotated (morphotaxa: two *Kophobelemnon* spp., one *Pennatula* sp., one *Protoptilum* sp., one *Virgularia* sp.), alongside 469 Cerianthidae sp. and 209 *Hyalonema* sp. individuals (**Table S3**). A shift in Pennatuloidea spp. composition was observed between 799 ± 3.5 m (SD) and 981 ± 1.4 m (SD) (transects 6–7), with assemblages transitioning from predominantly monospecific—dominated by *Kophobelemnon* msp1—to more compositionally mixed (Table 1; **Figure ****S1**).

**Table 1:** Density and distance statistics of Pennatuloidea spp. across transects alongside the *χ*^2^ statistics and *p*-values for the inhomogeneity tests (univariate and bivariate). Numbers in bold indicate the inhomogeneous transects. *Kophob*. msp1 = *Kophobelemnon* msp1; *Kophob*. msp2 = *Kophobelemnon* msp2; Penn. = Pennatuloidea spp.; ind. = individuals; nnd = nearest neighbour distance.

Transect ID	Mean depth ± SD (m)	Kophob. msp1	Kophob. msp2	Pennatula sp.	Protoptilum sp.	Virgularia sp.	Penn. spp. density (ind. m^−2^)	Min. NND (m)	Mean NND (m)	Univariate Inhomogen. χ^2^	*p*-value	Bivariate inhomogen. χ^2^	*p*-value
1	671 ± 3.5	32	0	0	0	0	0.6	0.3	0.7	14.10	0.096	19.42	0.543
2	725 ± 3.5	29	0	0	1	5	1.2	0.1	0.4	28.01	0.452	34.29	0.174
3	748 ± 1.4	63	0	0	0	2	3.1	0.1	0.3	**46.82**	**0.007**	**52.00**	**0.004**
4	765 ± 2.1	33	0	0	0	0	1	0.1	0.5	33.91	0.176	19.01	0.630
5	794 ± 2.8	60	0	0	1	3	2.6	0.04	0.3	35.53	0.065	33.67	0.087
6	799 ± 3.5	31	0	0	0	0	0.9	0.2	0.5	31.09	0.191	**54.14**	**0.003**
7	981 ± 1.4	23	23	15	0	0	1.6	0.04	0.4	15.63	0.291	**10.59**	**0.025**
8	984 ± 2.8	18	14	4	0	0	0.8	0.1	0.5	**57.86**	**0.002**	31.52	0.279
9	991 ± 7.1	15	4	11	0	0	0.6	0.2	0.7	12.44	0.105	27.85	0.427
10	1000 ± 5.7	27	0	6	1	0	0.8	0.1	0.5	20.78	0.838	20.71	0.753

### Univariate point pattern analysis

**Fig. 2:**
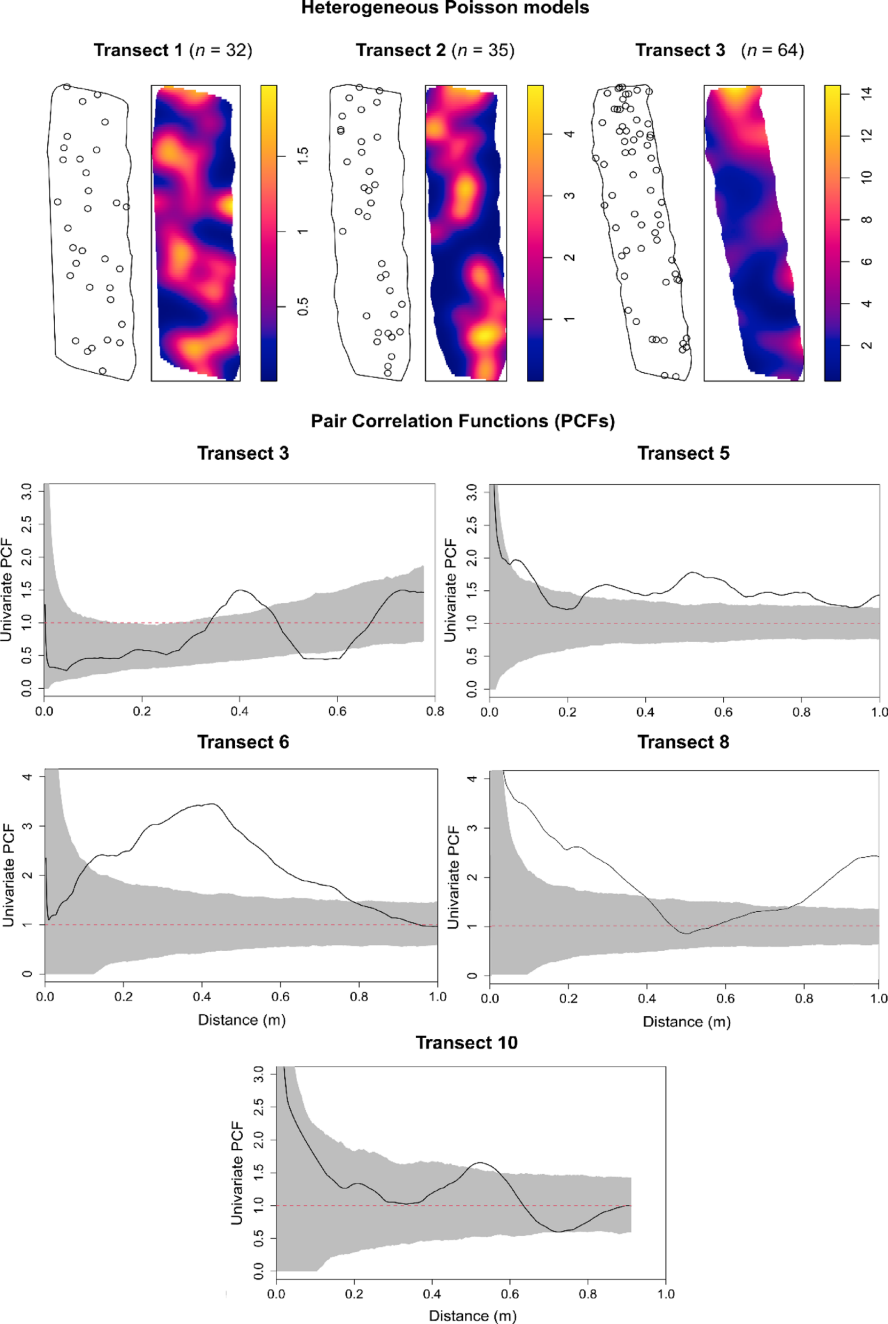
(Top) Examples of point patterns of Pennatuloidea spp. annotations across example transects with their respective heterogeneous Poisson models indicating habitat heterogeneity (kernel size = 1). A full catalogue of univariate point patterns is available in the Supplementary Information (Figure S2). (Bottom) Univariate PCF results for the four transects showing deviations outside the simulation envelope. *X*-axis: interpoint distance (m); *y*-axis: PCF = 1 (red dotted line) indicates Complete Spatial Randomness (CSR), PCF > 1 indicates clustering, PCF < 1 indicates overdispersion. The grey shaded area indicates simulation envelopes from 999 Monte Carlo simulations of CSR.

Different densities and spatial arrangements of Pennatuloidea spp. were observed across transects (Table 1; Figs. 2, S4). Each transect was assessed for inhomogeneity, with transects 1–2, 4–7, and 9–10 emerging as homogeneous and transects 3 and 8 emerging as inhomogeneous (Table 1). Point patterns were tested using the homogeneous/inhomogeneous PCF function, accordingly.

From the PCF plots, which quantify the density of sea pens at a growing distance *r* from a focal point, it can be observed that transects 3, 5, 6, and 10 had excursions outside the simulation envelope at distances ≥ 0.3 m, showing a clustering trend (Fig. 2). Transect 8, on the other hand, exhibited clustering at 0–0.4 m and 0.8–1 m distance. Transect 3 had a further excursion from the envelope at ca. 0.6 m, exhibiting an overdispersed pattern. The nature of the drivers was consistent across transects, with both clustering and overdispersion most likely driven by habitat heterogeneities with and without dispersal-limited reproductive processes (Table 2, S4–S5). These patterns were evidenced by the heterogeneous Poisson model (transects 3, 5, 6, and 8), indicating habitat associations, and inhomogeneous Thomas cluster model (transect 10), indicating dispersal limitation on a patchy habitat (Table 2), emerging as the best-fitting options for our data (transect 3: *p*_*d*_ = 0.844; transect 5: *p*_*d*_ = 0.998; transect 6: *p*_*d*_ = 0.556; transect 8: *p*_*d*_ = 0.342; transect 10: *p*_*d*_ = 0.639). Transects 1–2, 4, 7, and 9 showed no departure from Complete Spatial Randomness (CSR) (**Figures S5–S6**, **S8–S9**).

### Bivariate point pattern analysis

**Fig. 3:**
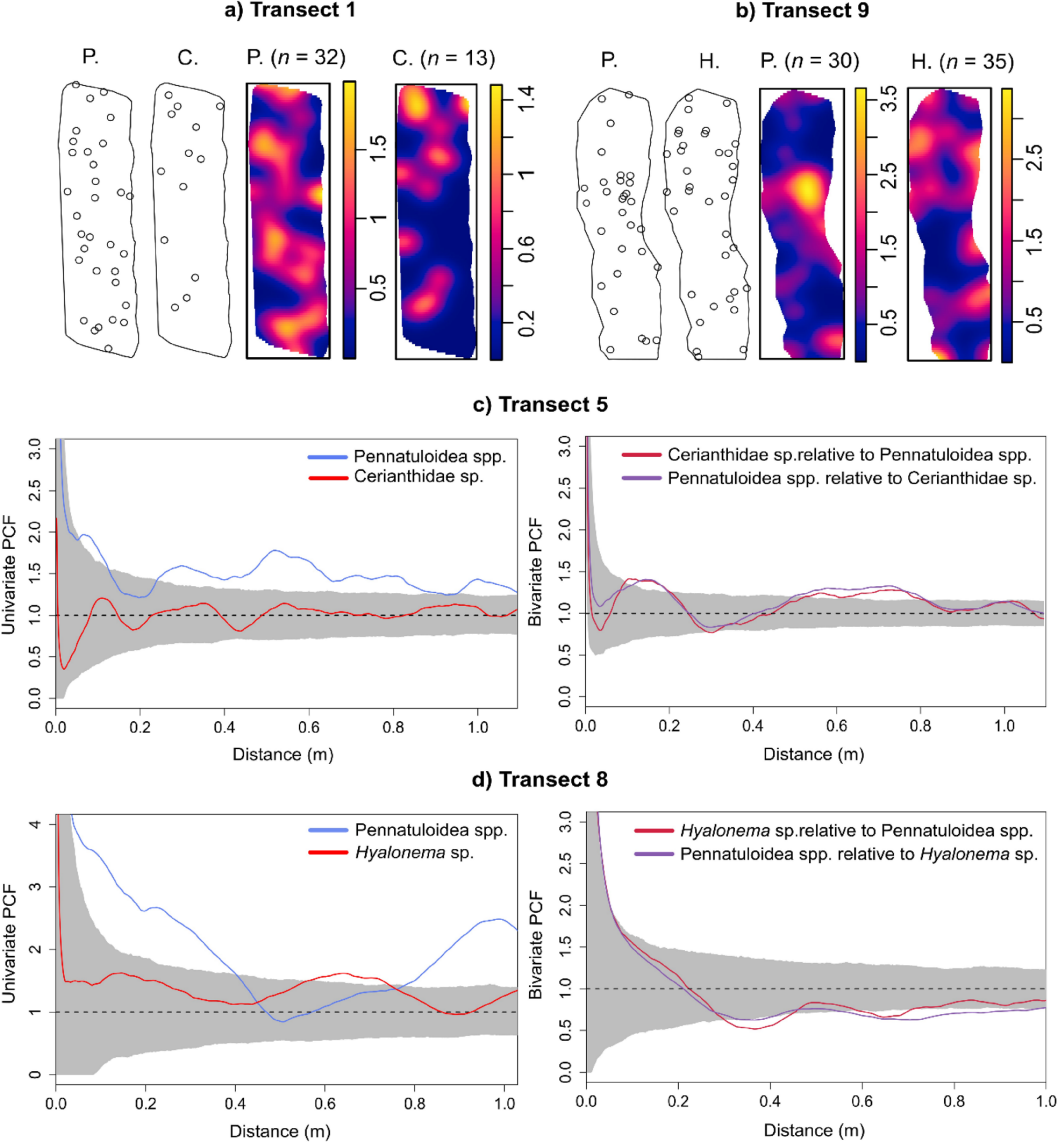
(**a**–**b**) Bivariate point patterns of Pennatuloidea spp. (P.) and Cerianthidae sp. (C.) or *Hyalonema* sp. (H.) in transects 1 (depth: 671 ± 3.5 m, SD) and 9 (depth: 991 ± 7.1 m, SD) with their respective heterogeneous Poisson models (kernel size = 1). (**c**–**d**) Multitype PCF of two example transects, transect 5 (794 ± 2.8 m, SD) and transect 8 (984 ± 2.8 m, SD). The grey shaded area indicates the simulation envelope (CSR: PCF = 1; clustering: PCF > 1; overdispersion: PCF < 1). Note that the univariate patterns in transect 5 follow similar patterns at different magnitudes, in contrast to transect 8 which shows notably different univariate patterns, but similar bivariate patterns.

Different combinations of taxa were observed across transects, with Pennatuloidea spp. occurring in conjunction with Cerianthidae sp. or *Hyalonema* sp. The spatial distributions of Pennatuloidea spp. and Cerianthidae sp. appeared to overlap, on average (Figs. 3a, S3).

A less obvious pattern emerged between Pennatuloidea spp. and *Hyalonema* sp. In most cases, the two exhibited little spatial overlap (Figs. 3b, S4). A statistically significant positive correlation was found between the transect-derived densities of Pennatuloidea spp. and Cerianthidae sp. (Kendall: *r*_*τ*_ = 0.69, *p*-value < 0.001), but not between Pennatuloidea spp. and *Hyalonema* sp. (Kendall: *r*_*τ*_ = 0.4, *p*-value = 0.483). Each transect was assessed for inhomogeneity, with transects 1–2, 4–5, and 8–10 emerging as homogeneous and transects 3, 6, and 7 emerging as inhomogeneous (Table 1). The bivariate point patterns were tested using the homogeneous/inhomogeneous multitype PCF function, accordingly.

A general clustering trend emerged between Pennatuloidea spp. and Cerianthidae sp., with the interorganism distance at which the pattern occurred varying across transects (Figs. 3c, S5–S7). When bivariate distributions are similar across taxa (Fig. 3c–d), as shown by Pennatuloidea spp. and Cerianthidae sp., this indicates mutual processes^35^. A mutual association due to a heterogeneous environment was evidenced by the heterogeneous Poisson models centred on the density of Cerianthidae sp. (transect 3: *p*_*d*_ = 0.960; transect 4: *p*_*d*_ = 0.104; transect 6: *p*_*d*_ = 0.894) and on the joint density of Pennatuloidea spp. and Cerianthidae sp. (transect 5: *p*_*d*_ = 0.816) emerging as the best fit for our data (**Table S6**). The best-fit heterogeneous Poisson models were similar for transects 3 and 5, despite the two being centred on the density of different taxa; therefore, limited conclusions can be drawn for these transects. The mutual habitat associations led to small-scale overdispersion between Pennatuloidea spp. and Cerianthidae sp. at distances < 0.3 m (transects 3–5; **Figures S6–S7**), with excursions outside the simulation envelope only occurring in transect 4. For transects 3–5, the distribution patterns exhibited by the bivariate PCFs did not reflect those of the univariate PCFs, suggesting that the overdispersion was likely not due to mutual habitat associations but rather local competition or, possibly, allelopathy.

A larger-scale overdispersion trend was observed between Pennatuloidea spp. and *Hyalonema* sp. (transects 8–10), with the interorganism distance at which the pattern occurred varying across transects (Figs. 3, S8–S9). To determine the ecological drivers of overdispersion in bivariate patterns with categorical labels (i.e., ‘Pennatuloidea’, ‘Cerianthidae’, ‘*Hyalonema’*), inferences were drawn based on a visual assessment of PCFs (Fig. 3d). The univariate PCFs of Pennatuloidea spp. and *Hyalonema* sp., respectively, did not show any signs of overdispersion for transects 8–10, suggesting that overdispersion only occurred in bivariate analyses, indicative of an intertaxa interaction. The difference between the univariate and bivariate patterns suggests that the driver was not an overdispersed mutual habitat association, but rather intertaxa competition^52^. In transect 8, specifically, the overdispersion was sufficiently strong to show evidence of statistically significant nearest-neighbour segregation (Χ^2^_2_ = 6.12, *p* = 0.0469), with further evidence of *Hyalonema* sp. segregation relative to Pennatuloidea spp. (Χ^2^ = 5.90, *p* = 0.0151). A complete summary of distance statistics for bivariate point patterns is presented in **Table S7**, where a closer spatial arrangement between Pennatuloidea spp. and Cerianthidae sp. is inferred relative to Pennatuloidea spp. and *Hyalonema* sp.

## Discussion

### Intrataxon clustering at the centimetric scale

Univariate point pattern analysis revealed a tendency among sea pens to cluster at distances ≥ 0.3 m, suggestive of habitat associations and dispersal limitation. While not statistically significant for all transects (5/10), the pattern demonstrates non-random (univariate) spatial organisation at the fine scale. This suggests that sea pens may show non-random distributions when occurring in fields, justifying further research over larger extents and across multiple sea pen fields. Given the centimetric scale at which the spatial distribution of sea pens was investigated, very few validated explanations for the observed clustering exist in the literature. At such small spatial scales, and in the context of octocorals, it is unknown how much individuals disrupt the water flow by inducing turbulence, and if sufficient resistance is exerted to influence proximal colonies’ feeding opportunities. Although it has been shown that a scleractinian CWC colony with an average diameter and height of 14 cm and 12 cm, respectively, can reduce the water flow by 70% in the first 20 cm of its wake^53^, the sea pen colonies in our study were generally smaller (7 cm mean height above the seafloor) and, most importantly, more flexible. Therefore, with the current state of knowledge, it is impossible to confirm or discount the possible influence of microscale flow dynamics on the observed clustering. This could be obviated with the construction of Computational Fluid Dynamic (CFD) models, which allow for the robust description and prediction of particle transport and deposition in a defined 3D structure based on mathematical models^54^. Moreover, the absence of oceanographic datasets at such fine spatial scales—either for the study area or for ecologically comparable sites—limits our ability to assess the hydrographic/oceanographic modification around topographic derivative variations such as slope, rugosity, and aspect at the centimetric scale, and thus to evaluate their potential influence. Additional factors, such as microtopography, may also contribute to the observed spatial patterns. Microtopography itself is likely shaped by physical processes, the presence of benthic fauna, or an interaction of both. Its role and underlying drivers could be investigated through nodule elevation analyses^55^ based on 3D reconstructions, as well as by comparing transect models with and without associated fauna. However, as our study design did not permit such comparisons, we are unable to draw conclusions regarding the influence of microtopography in sedimentary habitats at these fine spatial scales.

Although limited research exists on the larval phase of sea pens^56,57^, larval behaviour and settlement cues may be implicated in the observed clustering. In this study, reproductive-type clusters were identified in transect 10, with non-significant patterns suggestive of reproductive clusters in transects 3 and 5, the radius of which varied, reflecting possible different reproductive events^58^, possibly modulated by currents^59^. The impact of habitat heterogeneities varied across transects. Habitat heterogeneity was modelled as a significant driver for transect 6, while for transects 3 and 5, the impact was weaker, indicated by the value of the PCFs which indicates the strength of underlying processes (Fig. 2). Research on soft-sediment systems shows that permanent meiofauna play a role in the site selection by benthic macrofaunal larvae^60^, alongside the presence of sediment-associated microorganisms and microbial metabolites^61^. Release of chemical compounds also mediates larval settlement in cnidarians^62^ and soft-sediment systems^63^, with ammonium ions implicated in the larval settlement of some benthic taxa^64,65^. As enhanced ammonium efflux rates have been documented in association with sea pen presence^66^, the possible effects on conspecific larvae warrant investigation. Some cnidarian larvae release a compound that induces metamorphosis in conspecific larvae^67^, opening the possibility that adult sea pens may promote conspecific larval settlement through the release of unknown cues. Sea pen ‘mobility’ adds a further layer of complexity, although most observations seem to describe dislodgement under strong currents rather than active movement (e.g., ^68–70^). As no evidence exists pertaining the role of settlement cues or mobility in relation to sea pen assemblages or other soft-sediment octocorals, the drivers of the observed clustering remain speculative, highlighting a substantial lack of knowledge about sea pen life history traits.

### Intertaxa spatial distributions at the centimetric scale

When investigating the distributions of Pennatuloidea spp. and Cerianthidae sp., a general clustering trend emerged, most likely a mutual habitat association with habitat heterogeneities, e.g., topographical variation of the substratum^35^. The interorganism distance at which the pattern occurred varied across transects, possibly suggesting different underlying abiotic conditions. This clustering trend, while not statistically significant for all transects (4/6), demonstrates non-random (bivariate) spatial organisation at the fine scale (< 1 m), further supported by the statistically significant positive correlation found between the densities of Pennatuloidea spp. and Cerianthidae sp. Relative to Pennatuloidea spp., Cerianthidae sp. showed a greater variety of patterns, suggestive of a greater variety of underlying driving processes. Some of such patterns were relatively random (e.g., transects 4–5; **Figures S6–S7**), as suggested by the lack of pronounced excursions outside the simulation envelope, while others showed strong clustering (transect 3; **Figure S6**), likely caused by associations with habitat heterogeneities. Mutual habitat associations likely underlie the bivariate clustering in transects 5–6 (**Figure S7**), as the univariate patterns were similar and so were the bivariate counterparts. Mutual habitat association also likely explains the patterns in transects 3–4 (**Figure S6**), where it led to small-scale repulsion at < 0.15 m and < 0.3 m distances, respectively, as seen through the overdispersion in the bivariate pattern. However, crucially, overdispersion did not show in the univariate patterns, suggesting local competition or, possibly, allelopathy, the latter documented in numerous octocoral species^71–73^. Research in the NE Atlantic^48^ has documented assemblages of *K. stelliferum*—believed to be the dominant sea pen morphospecies in our study (**Figure ****S1**)—and a cerianthid anemone co-occurring in association with mud and muddy sand substrata. As in the case of sea pens clustering with sea pens, we suggest larval settlement cues as a possible driver. Although little is known about the larval phase of sea pens, in the Pacific Arctic, larvae of *Cerianthus* sp. have only been detected close to conspecific adult populations^74^, suggesting either recent spawning from that population or larval retention^74^. Larval behaviour, coupled with local hydrodynamics, can sometimes result in much shorter dispersal distances than predicted by larval duration, favouring propagule retention near their spawning grounds^59^. The faunal spatial patterns in transect 6, unlike those in transects 3 and 5, were found to be best modelled when centred on the density of a single taxon (i.e., Cerianthidae sp.), indicating that the bivariate relationship was asymmetric, possibly suggestive of a subtle facultative process by Cerianthidae sp. As facilitation by one species is a unilateral process, it would result in different bivariate PCFs^75,76^, with the facilitated taxon clustered around the facilitating taxon. The absence of this pattern from the bivariate PCFs, which were similar, alongside the fact that it was observed in only one transect, suggests that the putative facilitative processes did not exert a strong influence on the spatial arrangement of the investigated taxa. Therefore, it may be that the respective larvae of the two taxa ‘follow’ cues released by members of their own taxon, resulting in large clusters of the two.

Regarding the distributions of Pennatuloidea spp. and *Hyalonema* sp., a general overdispersion trend emerged at distances > 0.3 m, demonstrating non-random (bivariate) spatial organisation at the fine scale (< 1 m). This is further supported by additional evidence of statistically significant spatial segregation between the two taxa. Relative to Pennatuloidea spp., *Hyalonema* sp. showed a greater variety of patterns, likely indicative of associations with habitat heterogeneities. Transects 8–10 (**Figures S8–S9**) showed insignificant small-scale clusters; however, given their lack of statistical significance and the small scale, inferences of their underlying processes are limited. Transects 8–10 further exhibited bivariate overdispersion differing from that in the univariate patterns, suggesting intertaxa competition. Transect 9 showed a consistently stronger effect on *Hyalonema* sp. relative to Pennatuloidea spp., indicating that *Hyalonema* sp. may be more affected by such competition. The different univariate PCF distributions—indicative of different underlying processes—coupled with the similar bivariate distributions suggest that the two taxa compete. One possible explanation is ecological niche overlap. Both Pennatuloidea spp. and *Hyalonema* sp., along with the zoanthids observed on the stalk of the latter (Fig. 1j), feed on suspended particles and occupy comparable trophic positions^77,78^, despite the anomalous δ^15^N enrichment frequently documented in deep-sea hexactinellid sponges, including *Hyalonema* spp.^77^. While such enrichment may indicate a diet enriched in nitrogen-heavy sources^79^, it could also result from starvation events^80^, during which a greater proportion of nitrogen used in protein synthesis originates from catabolic rather than anabolic processes, and waste excretion preferentially eliminates the lighter ^14^N isotope^81^. Starvation events are consistent with the prolonged, rhythmic contraction–expansion cycles observed in deep-sea *Hyalonema* species^80,82^. Alternatively, or in addition, competition for limited substrate may also contribute to the observed overdispersion. Sponges, including hexactinellid species, are known to compete intensely with non-sponge taxa for space and are highly proficient in asexual propagation and regeneration following partial mortality^83–85^. Many of their competitive interactions are chemically mediated, with compounds produced either by the sponges themselves or by their symbiotic partners^83^. Evidence for similar interactions has also been observed in *Hyalonema* species^86^. Furthermore, sponges can significantly influence substratum stability and modify its suitability for colonisation by other organisms^87^—an ecological role that sea pens may also fulfil, potentially exerting analogous influences. Sea pens, too, may engage in chemically mediated interactions with the surrounding fauna^71,88,89^, influencing the composition and dynamics of the benthic community.

### On the significance to VME conservation and MCZ management

Understanding the fine-scale spatial distribution of VME indicators is crucial for effective VME management. Although regional planning tends to rely on broad-scale (100–1000s of km) bioregional frameworks^90^, finer-scale information (10–100s of m) is necessary for local implementation and for ensuring that variation within broad-scale habitats is represented in protected area networks^90^. Concerns exist about the reliability of broad-scale habitat categories with respect to the representation of biodiversity therein and the determination of conservation priorities^90^. Particularly in deep-sea settings, where quantitative data are scarce, discrepancies have emerged between the smallest units of management interest (100–1000s of km^2^) and the true size of VME habitats (< 1 km^2^)^91^. From a management perspective, fine-scale data are essential to create a representative baseline picture of the conservation area, enabling accurate long-term monitoring and interpretation of ecosystem dynamics^92^. Research in NE Atlantic submarine canyons has shown that the centimetric scale provides useful information to predict the structural and functional diversity of deep-sea benthic fauna^93^, with fauna-mediated processes disproportionately contributing to the observed variability^93^.

**Fig. 4:**
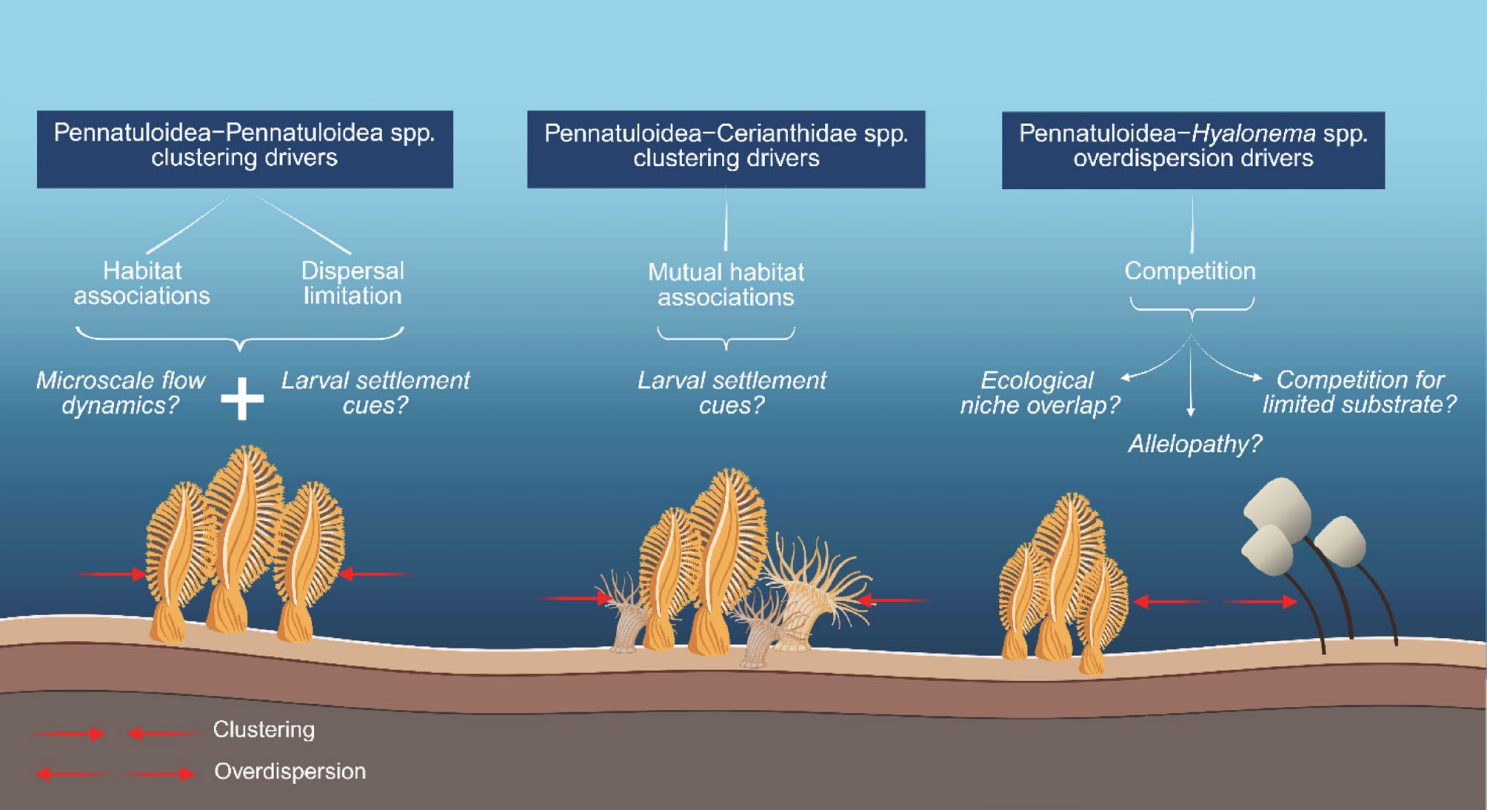
Schematic illustration of the possible drivers of the observed spatial patterns at the univariate and bivariate level. Created with BioRender.com.

Our study demonstrates that sea pen fields may not be randomly arranged in nature, highlighting the role of spatial pattern quantification within these habitats as an informative method for both their characterisation and the identification of distributional drivers (Fig. 4). We also demonstrate the importance of community composition and how this may affect VME characteristics, so that monospecific sea pen fields may well differ in their spatial arrangement and drivers from mixed-species fields in the NE Atlantic. This highlights the importance of our study in promoting a multiscale understanding of benthic ecosystem structure, such knowledge being critical for effective management of deep-sea benthic fauna. If we are to advance our understanding of deep-sea ecosystem functioning and of the mechanisms driving the structural and functional diversity of deep-sea benthic communities, particular attention must be devoted to small-scale heterogeneity and the causative processes acting on that scale^93^. By doing so, we objectively demonstrate that sea pens cluster with members of the same taxon and tube-dwelling anemones, a useful criterion for delineating sea pen fields and ‘sea-pen and burrowing megafauna communities’ VMEs.

## Limitations and future research

The present study is largely experimental in nature, applying SPPA to opportunistic ROV data on deep-sea sea pen assemblages. A key limitation of this and any future studies relying on opportunistic video data is the limited control over the geometry of observation windows for SPPA purposes. Typical ROV monitoring surveys result in observation windows that are substantially longer than they are wide^45^. SPPA research, however, tends to rely on windows that are either square or wider than the transects produced in this study^32,33,41^, as the spatial scale relevant to SPPA equals approximately half the length of the shortest window edge, in this case, the width of transects^94^. Whilst the pooling of multiple point patterns within a “hyperframe” could obviate the slender nature of ROV-derived transects and its impact on sample size, true replicates would be required^32^. Unfortunately, this was not possible in the present study, as the video transect data on which our analyses relied commenced at the thalweg of the Dangaard Canyon and ascended along its northern flank. The ability of sea pen species—such as *Pennatula aculeata*^95,96^, *P. rubra*^97^, *Protoptilum carpenteri*^98^, and *Virgularia mirabilis*^99^—and tube-dwelling anemones^100^ to fully withdraw into the sediment in the apparent absence of external stimuli presents an additional challenge, potentially influencing the observed spatial patterns. As such behaviour can only be documented through the deployment of multiple time-lapse cameras^95^—which were not available at our study site—and the resolution of the reconstructed transects was insufficient to determine the presence or absence of burrows, this factor could not be accounted for within the scope of the present study. Moreover, even if burrows had been discernible, it would not have been possible to determine the identity of the inhabiting organisms or if the burrows were indeed still inhabited. Lastly, the decision to group different sea pen morphospecies for the purpose of spatial analyses may have also affected the observed patterns to some extent. While focusing exclusively on the most abundant morphospecies could have produced results more directly applicable to conservation planning, it would have also reduced the number of usable transects from ten to five, given that a minimum of 30 points is required for SPPA^31^. However, as *Kophobelemnon* was by far the most abundant genus across all transects except transect 9—where *Pennatula* sp. colonies were more numerous than *Kophobelemnon* msp2, though not *Kophobelemnon* msp1—we anticipate that the impact of this decision on our results was negligible.

Despite such limitations, this work demonstrates that SPPA can uncover ecological patterns of poorly understood benthic fauna even when applied to opportunistic, suboptimal data. More specifically, this study demonstrates that SPPA can quantify biotic interactions on a continuous scale, determining at what scale the nature of a given interaction changes. This study further demonstrates that SPPA can be applied to transects, as evidenced also by Prado et al.^101^. Nonetheless, validation studies are required to confirm that results from photogrammetry transects are representative of wider benthic patterns. The approach adopted in this study is suitable for application to other ecosystem engineering fauna, e.g., xenophyophore fields and coral gardens^11^. Should ecological trends emerge from these pilot studies, more dedicated and rigorous investigations into the fine-scale spatial organisation of the organism of interest should be pursued, with data collected as focused photomosaics or as part of 3D photogrammetry surveys to address project-specific questions.

With respect to the present study, we suggest replication of the analyses at different locations in the canyon system using wider and longer observation windows (assuming a sufficient density of sea pens) to confirm or disprove the trends identified. To validate niche overlap as an overdispersion driver, stable isotope, elemental, and fatty-acid analyses in the Dangaard Canyon could detail the trophic relationships of the local benthic taxa. Furthermore, aquarium manipulations should be undertaken to investigate the larval phase of deep-sea sea pens and co-occurring species, alongside the cues mediating their settlement. Lastly, to better understand the influence of individual sea pens on microscale flow dynamics and particle flow paths in their vicinity, the construction of CFD models should be considered to assess the wake, and, thus, potential influence, of the flow surrounding single and multiple colonies^54^. The above would promote a more in-depth understanding of habitat selection by the investigated VME taxa. The resulting knowledge may afford valuable insights into their distribution, in turn improving the predictive power of habitat suitability models and our understanding of ecosystem functioning, thus informing the design of effective marine conservation zones.

## Conclusions

This study offers a quantitative analysis of the spatial organisation of deep-sea sea pens in a submarine canyon conservation zone (NE Atlantic), representing, to the best of our knowledge, the first in situ fine-scale quantification of univariate and bivariate spatial patterns within sea pen assemblages. At the intrataxon level, sea pens exhibit a tendency to cluster, most likely driven by attraction to habitat heterogeneities. However, the extent to which individual organisms may disrupt water flow at such small spatial scales—potentially affecting the feeding efficiency of neighbouring colonies—remains uncertain, as does the degree of microtopographic variability at the centimetric scale. In light of these unresolved factors, we propose larval settlement cues as a potential driver—or one of several possible drivers—of the observed spatial patterns. However, the potential influence of microscale flow dynamics and microtopographic variations cannot be discounted. At the intertaxa level, mutual habitat association with habitat heterogeneities—potentially including cues for larval settlement—may help explain the observed clustering, while ecological niche overlap, competition for limited substrate, and/or allelopathy are likely to account for the overdispersed patterns. Despite its limitations, this work demonstrates the benefits of spatial analysis techniques in the context of poorly understood taxa. The ecological implications of these findings raise new research questions requiring further hypothesis testing, paving the way for more comprehensive ecological and spatial investigations into deep-sea sea pen assemblages in support of their effective protection.

## Methods

### Study area

**Fig. 5:**
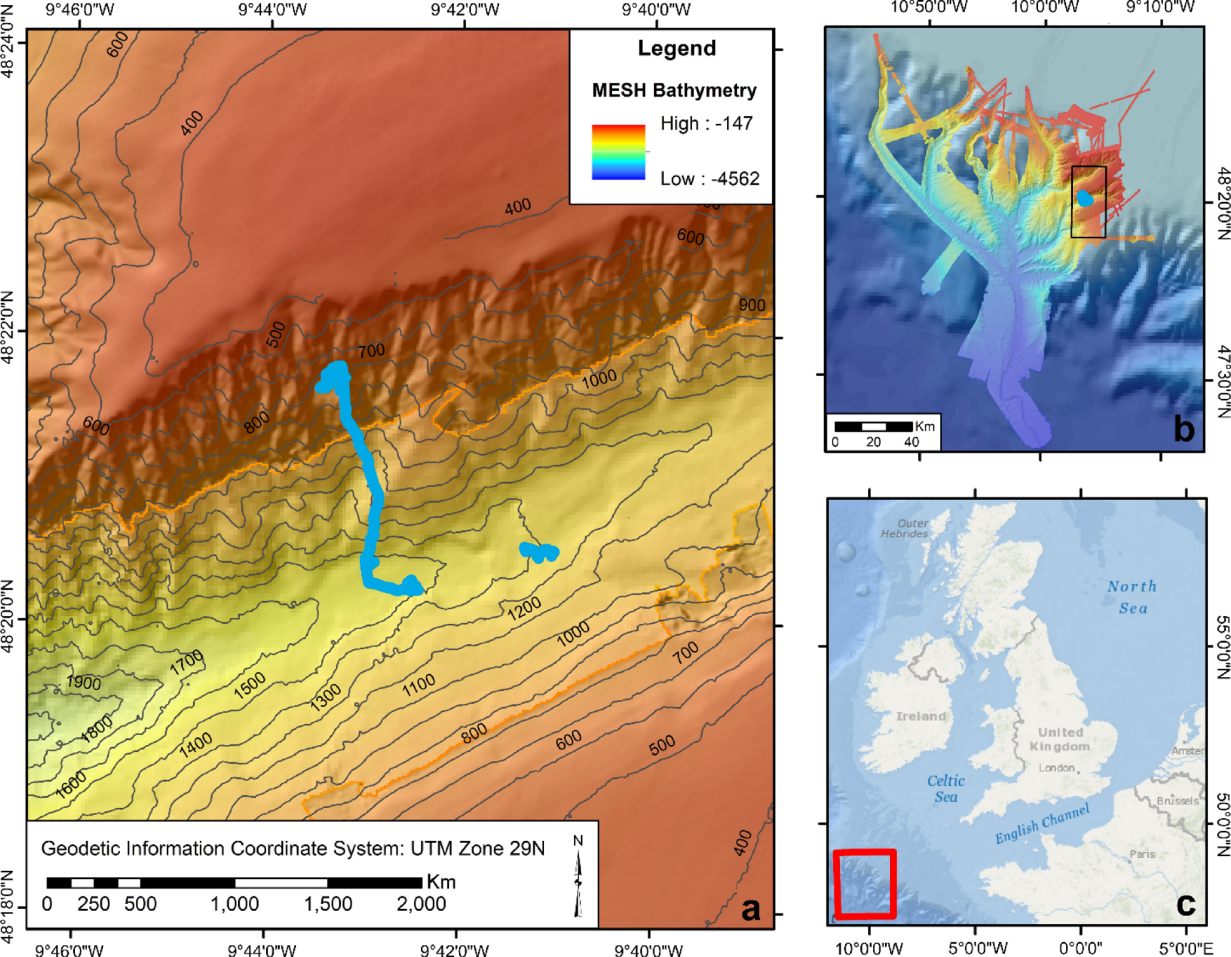
Close-up view of the northern flank of the Dangaard Canyon with the path of the ROV *Isis* (blue) (**a**). Inset (**b**) positions Dive 386 (blue dot) in the broader context of the Marine Conservation Zone (MCZ, black rectangle) and the Whittard Canyon system (coloured bathymetry). The MESH bathymetry interpretation key presented in the legend applies to both insets (a) and (b). In Inset (b), two other bathymetry layers are present: bathymetry data for the Whittard Canyon collected during cruises JC035, JC125, and JC237 by the National Oceanography Centre (NOC) and background GEBCO bathymetry^103^ in shades of blue. Inset (**c**) positions the study site (red box) within its broader geographical context. The map was produced in ArcMap v10.8.2 (ESRI, 2021).

Sea pen assemblages were explored in The Canyons MCZ, located within the Dangaard Canyon, a side branch of the Whittard Canyon system on the Celtic Margin (NE Atlantic; Fig. 5). The analysis was based on opportunistic benthic imagery collected by the ROV *Isis* during Dive 386 of the JC237 research expedition in August–September 2022, aboard RRS *James Cook*^102^.

### Image surveying

A video transect of 09:21 hours commencing from the thalweg of the Dangaard Canyon and ascending along its northern flank—at a constant speed of 0.2 knots and a constant altitude of 2 m—was conducted by the ROV *Isis* during Dive 386, covering depths between 671 m and 1367 m (Fig. 5a). Dive 386 was completed between 48°20’35.16” N, 9°42’54.18” W and 48°21’39.06” N, 9°43’24.46” W. The ROV *Isis* acquired high-definition videos and stills of the seabed, collected by the fixed-zoom, static, obliquely angled (22.5°) SCORPIO camera (Sony HDR-CX560v, Complementary Metal Oxide Semiconductor, CMOS, image sensor). The SCORPIO camera was set to collect one still image of the benthic environment for every 30 s of footage, resulting in 1203 images of 4672 × 2628 pixels; LED illumination was afforded by APHOS 16 LED units (Cathx Ocean). Two red lasers spaced 0.1 m were mounted either side of the SCORPIO camera to allow for scaling within the images. ROV navigation data from the Sonardyne Ultra-Short BaseLine (USBL) positioning system were extracted from records obtained from the Ocean Floor Observation Protocol (OFOP) software.

### Canyon flank scale

#### Image selection

The 30-second-interval SCORPIO images were grouped into 13 consecutive depth bins (50 m each), spanning from 686 m to 1328 m water depth. Within each bin, a minimum of five randomly selected, non-overlapping images were chosen to serve as replicates. Prior to image selection, depth-binned images were subject to visual inspection and stills containing exclusively the water column, focus aberrations, or elevated turbidity levels were excluded from the image selection process. Random replicates were selected from the retained images using the RANDBETWEEN() function in Excel Microsoft 365, resulting in a total of 104 images chosen for annotation.

#### Image annotation

Images were annotated manually in a randomised order using the BIIGLE 2.0 web annotation service^104^. A label tree based on the Standardised Marine Taxon Reference Image Database (SMarTaR-ID) was utilised^51^. SMarTaR-ID classifies organisms into separate morphospecies—i.e., species designated based on recognisable and unequivocal morphological attributes—by assigning Operational Taxonomic Unit (OTU) numbers over taxon names to organisms for which a lack of physical (preserved) specimens hinders conclusive taxonomic identification^51^. The standardised reference guide was implemented to increase comparability and interpretability of both benthic imagery and findings presented in this study. Owing to the obliquely angled nature of the SCORPIO camera, in a small subset of images where the terrain was less steep and, hence, not perpendicular to the camera angle, the water column was visible in the background. To accommodate for such cases, the effective area of the annotated portion of each frame was computed in BIIGLE. This was achieved by drawing a polygon around the well-lit portion of each image and by assigning it the lost and found SMarTaR-ID label, as no specific label was available for field of view calculation. Successively, an automatic laser point detection was performed to enable the scaling of each image and the computation of the visual footprint of the annotatable area (mean of 4.01 ± 0.24 m^2^, SE). Morphospecies density was calculated for each image to explore their vertical distribution along the canyon slope.

### Fine-scale structure-from-motion

#### Computation of 3D reconstructions

The entirety of the raw ROV video footage from Dive 386 (09:21 h) was visually inspected for sections containing a minimum of five closely distributed sea pen colonies, i.e., visible within the same frame. Upon locating five individual colonies within a frame, the corresponding time in the video was noted as the start of the section. When the number of sea pens visible dropped below five in a subsequent frame, the corresponding time was marked as the end of the section. Those sections were successively extracted using Quicktime 7 Pro (Apple Inc) and further divided into 10 m subsections for photogrammetry modelling. The 10 m cut off was obtained by calculating the X-Y-Z distance travelled by the ROV, as measured by the USBL positioning system (Sonardyne). A length of 10 m was deemed appropriate given the aggregating nature of sea pen taxa^105^ and the known accumulation of positioning error with increasing distance which can impact longer reconstructions^45^. Following visual inspection, a total of 78 subsections (i.e., transects) were deemed of sufficient resolution and quality (e.g., no sediment cloud, good lighting). Stills were extracted from each subsection at a rate of one frame per second to preserve ≥ 75% of image overlap (FFmpeg v5.1.3 “Riemann” libraries) and were cropped in IrfanView v4.62 to retain only the portions affording a clear and distinguishable view of the seabed. Cropped images were colour corrected for underwater light attenuation in Matisse 3D v1.5.0^106^ (channel saturation percentage = 0.6; **Figure S10**).

**Fig. 6:**
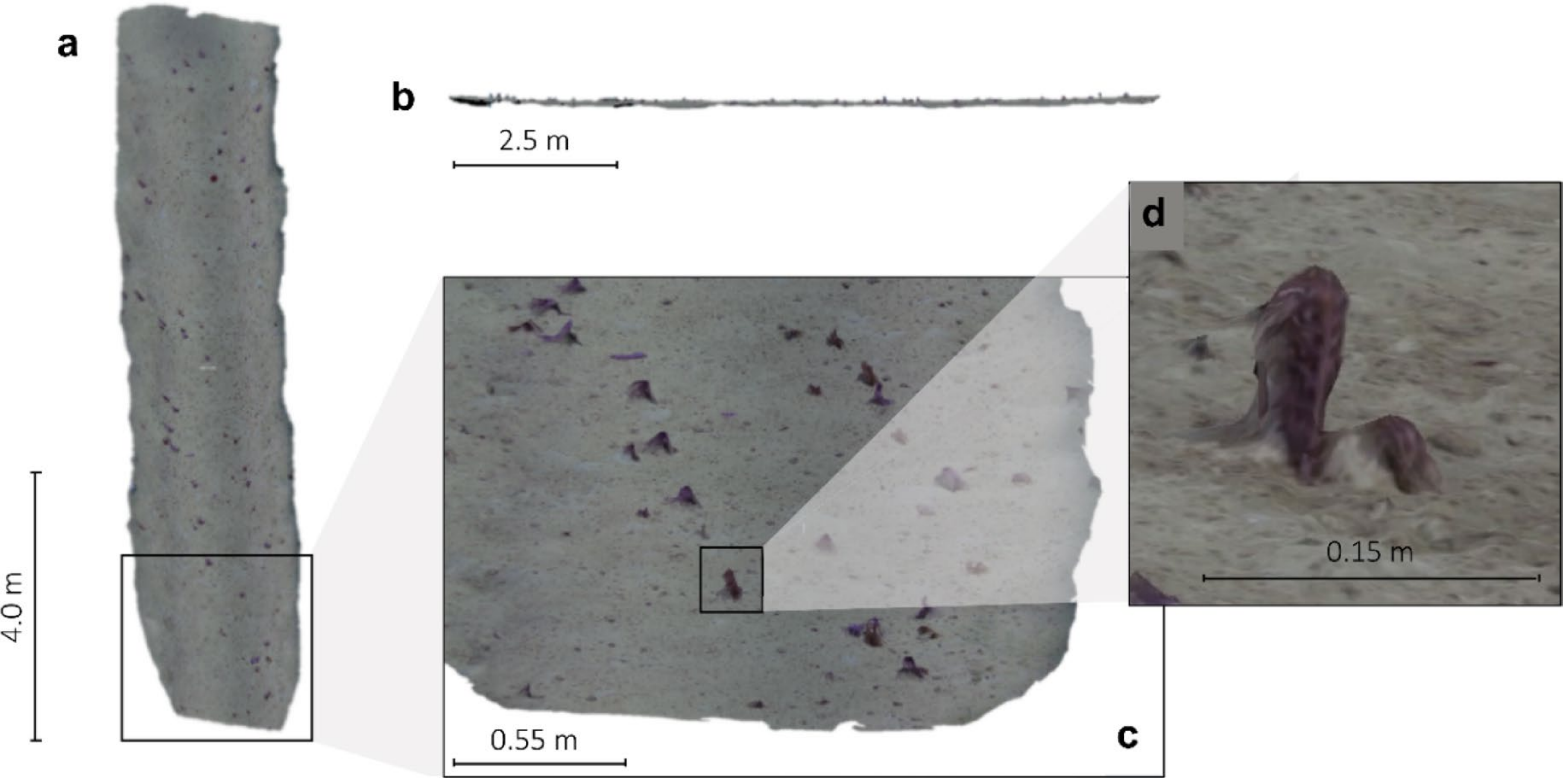
Example of three-dimensional reconstruction: (**a**) top-down view of transect 3; (**b**) left-side view of the orthorectified transect 3; (**c**) close-up, oblique view of transect 3; (**d**) close-up view of individual 3D-reconstructed sea pen (*Kophobelemnon* msp1, SM871). Each view is presented with their respective scale bar. Produced in Meshroom v2023.2.0^107^ and post-processed in CloudCompare v.2.12.4.

Pre-processed images were imported into the open-source 3D reconstruction software Meshroom v2023.2.0^107^ (see Supplementary **Figure S11** for a detailed workflow) and merged into textured mesh objects (.obj). Any image that failed to be feature-matched by the software was automatically discarded. Textured mesh objects were scaled using laser points present in the texture (Multiply/Scale in CloudCompare v2.12.14; Fig. 6a) and carefully inspected for mismatches, such as gaps or duplicate features, which could introduce pseudoreplication. No such aberrations were observed. Additionally, models were orthorectified to the perpendicular angle of the plane to minimise the influence of the broad-scale topographic relief (i.e., slope) on point pattern analysis, the latter not accounting for the Z axis (Δz < 1 m; Fig. 6b).

Photogrammetry was applied to frames extracted from randomly selected subsections until 14 transects were successfully reconstructed. The target number of photogrammetry transects had been set to 15, but the reconstruction of transects was successful in 14 cases only. Of these successful reconstructions, only transects containing a minimum of 30 univariate points (i.e., sea pen colonies; Fig. 6c, d) were retained to ensure sufficient statistical robustness^31^. This resulted in 10 transects, covering an area of 371.7 m^2^ and spanning a depth range of ca. 330 m, being used for spatial analysis (**Figures S12–S13**). These 10 reconstructions were generated from 4823 frames extracted from a total of four hours of ROV footage, with each transect consisting of 482.3 ± 38.6 images (SE), on average (**Table S8**). The mean length of the three-dimensional reconstructions was 11.16 ± 0.4 m (SE).

#### Morphotaxa annotation

The orthorectified reconstructions were imported into 3DMetrics v1.0.0^106^ for morphospecies position annotation. In 3DMetrics, three annotation groups were created: (i) Pennatuloidea spp., (ii) the most abundant non-Pennatuloidea taxon in each transect (either Cerianthidae sp. or *Hyalonema* sp.), and (iii) transect outline. The reason behind the decision to include only point data from one non-Pennatuloidea taxon in the second annotation group is that Cerianthidae sp. and *Hyalonema* sp. were never found in the same transect with Pennatuloidea spp. in comparable abundances: one was always abundant, while the other was either absent or scarce (**Table S3**). The transect outline was traced by constructing a detailed polygon around the perimeter of each transect, and its area was computed by selecting area as the annotation type in 3DMetrics. Positional data (point annotation) were acquired for all individuals/colonies belonging to the assessed morphotaxa. Morphotaxa annotations on each transect were ground-truthed using both the extracted frames employed for 3D model generation and raw ROV footage due to its higher quality and to address the occasional issue with camera orientation. Upon completion of the annotation process, each annotation group was exported as both a data table (.csv) and a shapefile (.shp) for analysis, and density estimates were calculated for each morphotaxon of interest.

### Spatial analysis

Spatial analyses were conducted in R v4.3.1. using packages spatstat and spatstat.explore^32^, with methods adapted from Price et al.^41^. Although a maximum extent of 10 m (i.e., the size of our observation window; see Sect. *Computation of 3D reconstructions* in the Methods) was selected, the spatial scale relevant to SPPA corresponds to approximately half the length of the shortest edge of the window—namely, the transect width^94^, which measured approximately 2 metres. Consequently, the effective extent of the SPPA was constrained to 1 metre. The analysis was conducted at a centimetric resolution (i.e., measurement precision^108^), enabling the detection of spatial patterns within a 1 m radius (0–1 m) of each focal point.

A selection of methods was implemented to describe and quantify the spatial point patterns of sea pens relative to (i) sea pens (univariate) and (ii) the most dominant non-sea-pen taxon (bivariate) in each transect. Mean nearest neighbour distance and minimum nearest neighbour distance were computed to describe the spatial distribution of Pennatuloidea spp., Cerianthidae sp., and *Hyalonema* sp. Successively, heterogeneous Poisson models (i.e., density plots of the organisms’ positions) were generated using a fixed-bandwidth kernel density estimate (k = 1)^[41]^. Univariate and bivariate point patterns were assessed for inhomogeneity, a form of first-order non-stationarity where the mean density of points varies across the study region^109^. The inhomogeneity of point patterns can be ascribed to terrain variables such as slope, aspect, rugosity, as well as fine-scale sediment properties including grain size, cohesion, and composition. Additionally, inhomogeneity can be caused by dispersal, biotic interactions, or mortality processes. Inhomogeneity was assessed through quadrat testing using the Monte Carlo statistical method, as the small sample sizes weakened the robustness of the chi-square (χ^2^) approach (with the chi-square statistic being the default in the quadrat test). A *p*-value > 0.05 was taken to indicate homogeneity^110^. Lastly, the Pair Correlation Function (PCF, *g(r)*), which quantifies the density of ecological objects at a non-accumulative growing distance *r* from a focal point^32^ (**Figure S14**), was utilised as a summary statistic of the point patterns. As the standard PCF assumes homogeneity, the inhomogeneous PCF *ginhom(r)* was used when the homogeneity assumption was violated. The inhomogeneous PCF *ginhom(r)* is a summary of the dependence between points in a spatial point process that fails to exhibit a uniform density of points^32^, akin to a non-parametric statistical test used for data with non-normal distributions. As part of the PCF computation, point patterns were compared with 999 Monte Carlo simulations of a Complete Spatial Randomness (CSR) pattern, with the 49th highest and lowest simulation values chosen to be the limits of the simulation envelopes, in line with previous work^35,111^. Any deviations above or below CSR (PCF = 1) suggest clustering (PCF > 1) or overdispersion (PCF < 1), respectively, with any deviations outside the simulation envelope indicating statistical significance. Ripley’s isotropic edge correction, implemented for rectangular and polygonal windows^112^, was applied to PCF computations. For bivariate point patterns, a multitype PCF was used instead, with the inhomogeneous version used for bivariate point patterns lacking homogeneity. For further details on SPPA, and PCFs in particular, the reader is directed to the Supplementary Information (Sect. 1).

When non-random patterns were identified in univariate SPPA (i.e., PCF > 1 or PCF < 1), a range of different models were constructed and fitted to the point data to infer the ecological drivers of the observed distributions. Models included the heterogeneous Poisson (relative to the *x*, *y*, and *x–y* directions, and the density of Pennatuloidea spp.’s points), homogeneous and inhomogeneous Thomas cluster (relative to the *x*, *y*, and *x–y* directions), soft-core process (with kappa values from the minimum allowed of 0.1 to the maximum allowed of 0.9), and hard-core process (relative to the *x*, *y*, and *x–y* directions) models^35^. Based on the best-fitting model, inferences were made about the likely drivers of each point pattern (Table 2). Given the absence of auxiliary environmental and biological data, model fitting was merely used to classify the most likely driver(s) as biotic (facilitation or competition) or abiotic (habitat associations with or without dispersal limitation).

**Table 2:** Ecological drivers, spatial patterns, and the most appropriate model choice. Adapted from Mitchell and Harris^35^.

Driver	Spatial pattern	Best modelled by	Refs.
Habitat association	Clustering (univariate)	Heterogeneous Poisson models	^35,111,113^
	Clustering (bivariate)	Heterogeneous Poisson models	^111,113^
	Overdispersion (univariate/bivariate)	Heterogeneous Poisson models	^114^
Facilitation	Clustering (bivariate)	Linked Thomas cluster models	^76,115^
Reproductive/dispersal processes	Clustering (univariate)	(Homogeneous) Thomas cluster models	^52,113,116^
Reproductive/dispersal processes with habitat associations	Clustering (univariate)	Inhomogeneous Thomas cluster models	^52,113,116^
Competition	Overdispersion (univariate/bivariate)	Hard-core (if there is no overlap of organisms within a given radius) and soft-core (if organism density is reduced) process models	^34,52^

A total of 999 simulations of each model were generated to produce the simulation envelope. The 49th highest and lowest simulation values were selected to be the limits of the simulation envelopes, in line with previous work^35,111^. Model selection was performed in two steps. First, each model was fitted to our data and the two models showing the lowest Akaike Information Criterion (AIC) scores within each model category (e.g., heterogeneous Poisson, etc.) were selected for further checks. Second, the goodness-of-fit of each of these models was assessed using the Diggle’s goodness-of-fit test^117^. This test performs hypothesis assessments for the goodness-of-fit of a point pattern dataset in relation to a point process model based on Monte Carlo simulations (999) from the model and determines the total squared deviation between the observed pattern and the simulated pattern across the studied distances^118^. As recommended by the developers of the Diggle’s test function in R (‘dclf.test’), the *L* function was selected as the summary function^119^. Although the Diggle’s test statistic, *p*_*d*_, does not strictly test whether a model should be accepted or rejected, it indicates whether the test calculation for the observed data are within the range of the stochastic realisation of the null hypothesis^120^. In line with previous work, the *p*_*d*_ value was interpreted as being indicative of a good fit the closer it got to a value of 1^35^.

With respect to bivariate SPPA, the following approach was adopted. For clustered distributions, a joint point pattern was created and fitted with three separate heterogeneous Poisson models: (1) one based on the density of sea pen colonies, (2) one on the density of the dominant non-sea-pen taxon, and (3) one on the joint density of the two taxa. In the instance of models (1) or (2) emerging as the best fit, facilitation was inferred, with aggregations of the facilitated taxon centred on the facilitating taxon. In the instance of model (3) best-fitting the data, mutual habitat association was inferred, with the clustering of the two taxa independent of the distribution of the other taxon. For overdispersed patterns, ecological inferences were drawn based on a comparison between the univariate PCF plots of each taxon and the corresponding bivariate PCF plots. Should the univariate PCF plots of the two taxa display overdispersion at the intrataxon level, occurrence of an overdispersed trend in the bivariate PCF plots would not be interpreted as the two taxa repulsing each other. Conversely, should the univariate PCF plots show no overdispersion at the intrataxon level, emergence of overdispersion in the bivariate PCF plots would be interpreted as the two taxa repulsing each other.

## Supplementary Information

Below is the link to the electronic supplementary material.

Supplementary Material 1

## Data Availability

Due to the large size of the generated 3D models, all data are available from the corresponding authors upon reasonable request.
