# Supplementary material for: Fine-scale spatial organisation of deep-sea sea pens in a NE atlantic submarine canyon conservation area
Source: Sci Rep. 2025 Aug 11;15:29332. doi: 10.1038/s41598-025-13327-2 (PMC12339986; doi:10.1038/s41598-025-13327-2)
Supplement: Supplementary file 1 — Supplementary Material 1 [file 41598_2025_13327_MOESM1_ESM.docx]

**Supplementary Information for “Fine-scale spatial organisation of deep-sea sea pens in a NE Atlantic submarine canyon conservation area”**

Irene Susini^*1,2^, Loïc Van Audenhaege^3^, David M. Price^4^, Tabitha R. R. Pearman^3^, Emily G. Mitchell^5^, Veerle A. I. Huvenne*^3^

^1^ Ocean and Earth Science, University of Southampton Waterfront Campus, Southampton SO14 3ZH, United Kingdom

^2^ School of Biological and Marine Sciences, University of Plymouth, Drake Circus, Plymouth PL4 8AA, United Kingdom

^3^ National Oceanography Centre, European Way, Southampton SO14 3ZH, United Kingdom

^4^ Institut de Ciències del Mar, Consejo Superior de Investigaciones Científicas (ICM-CSIC), Passeig Marítim de la Barceloneta, 37-49, 08003 Barcelona, Spain

^5^ Department of Zoology, University Museum of Zoology, University of Cambridge, Cambridge, United Kingdom

[*irene.susini@plymouth.ac.uk](mailto:*irene.susini@plymouth.ac.uk), [irenesusini3@gmail.com](mailto:irenesusini3@gmail.com), [vaih@noc.ac.uk](mailto:vaih@noc.ac.uk)


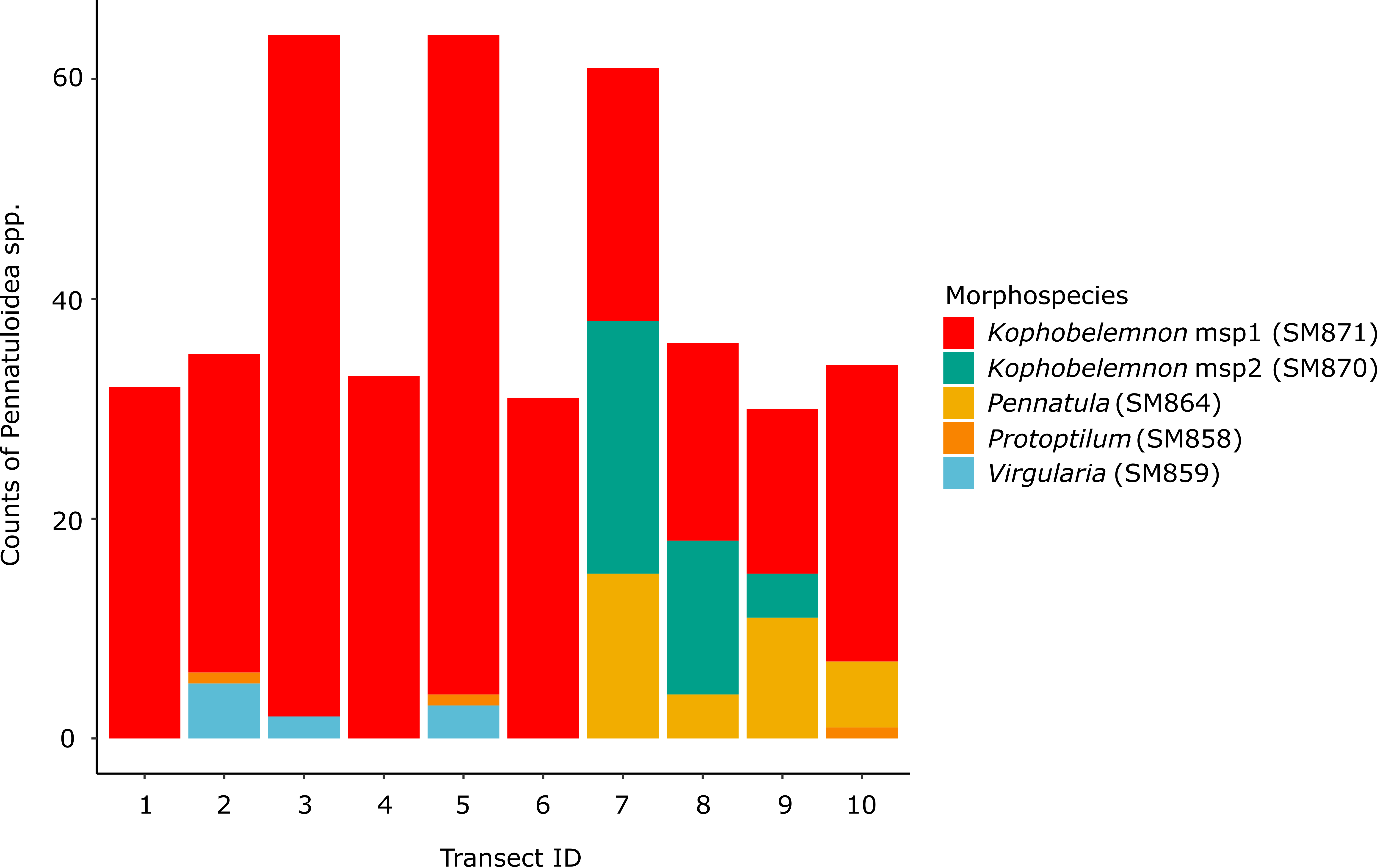


**Figure S1.** Pennatuloidea morphospecies distribution across photogrammetry transects. Mean water depth ± SD of each transects: 1) 671 ± 3.5 m; 2) 725 ± 3.5 m; 3) 748 ± 1.4 m; 4) 765 ± 2.1 m; 5) 794 ± 2.8 m; 6) 799 ± 3.5 m; 7) 981 ± 1.4 m; 8) 984 ± 2.8 m; 9) 991 ± 7.1 m; 10) 1,000 ± 5.7 m. Morphospecies classification was performed as per the SMarTaR-ID image repository.


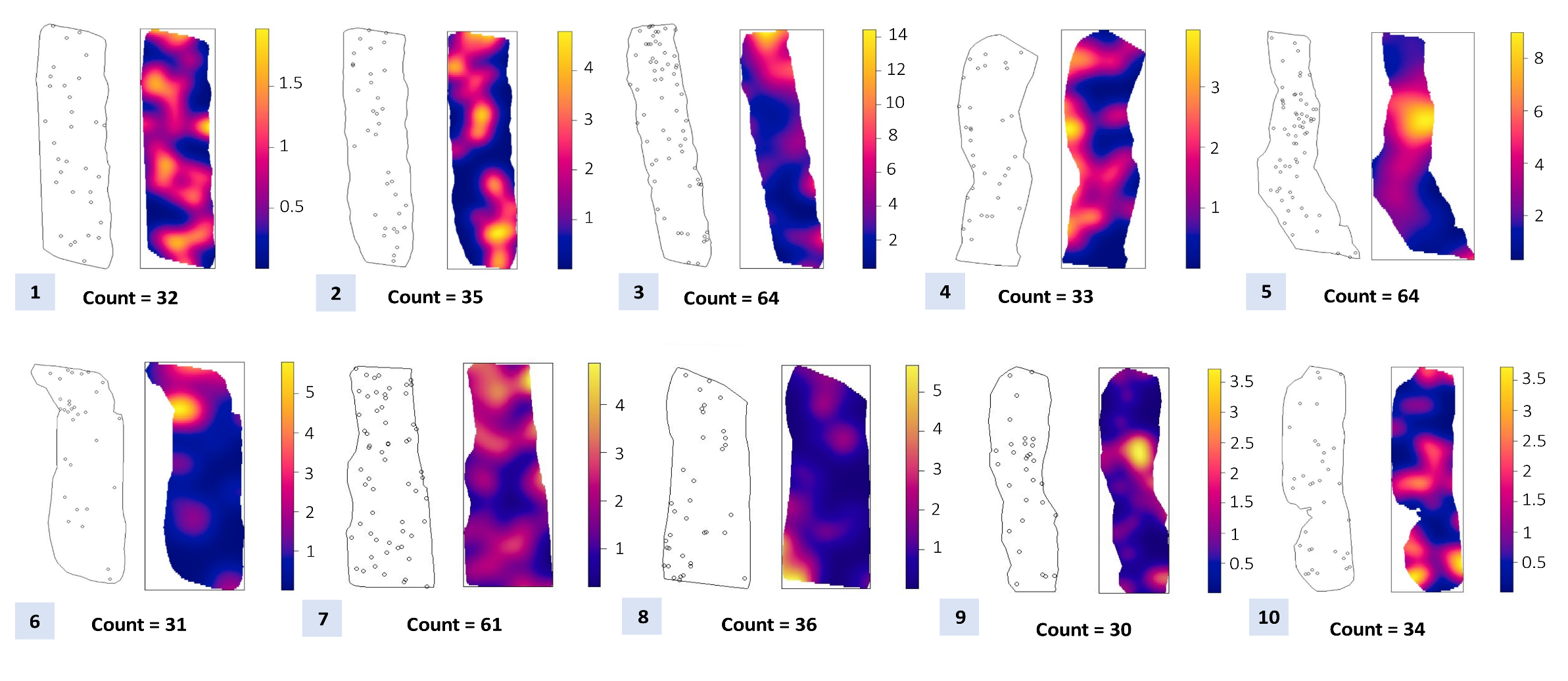


**Figure S2.** Point patterns of Pennatuloidea spp. annotations across transects with their respective heterogeneous Poisson models (kernel size = 1). Total Pennatuloidea spp. counts and transect identifying numbers are reported for each transect. Note the varying colour scale amongst the different plots.


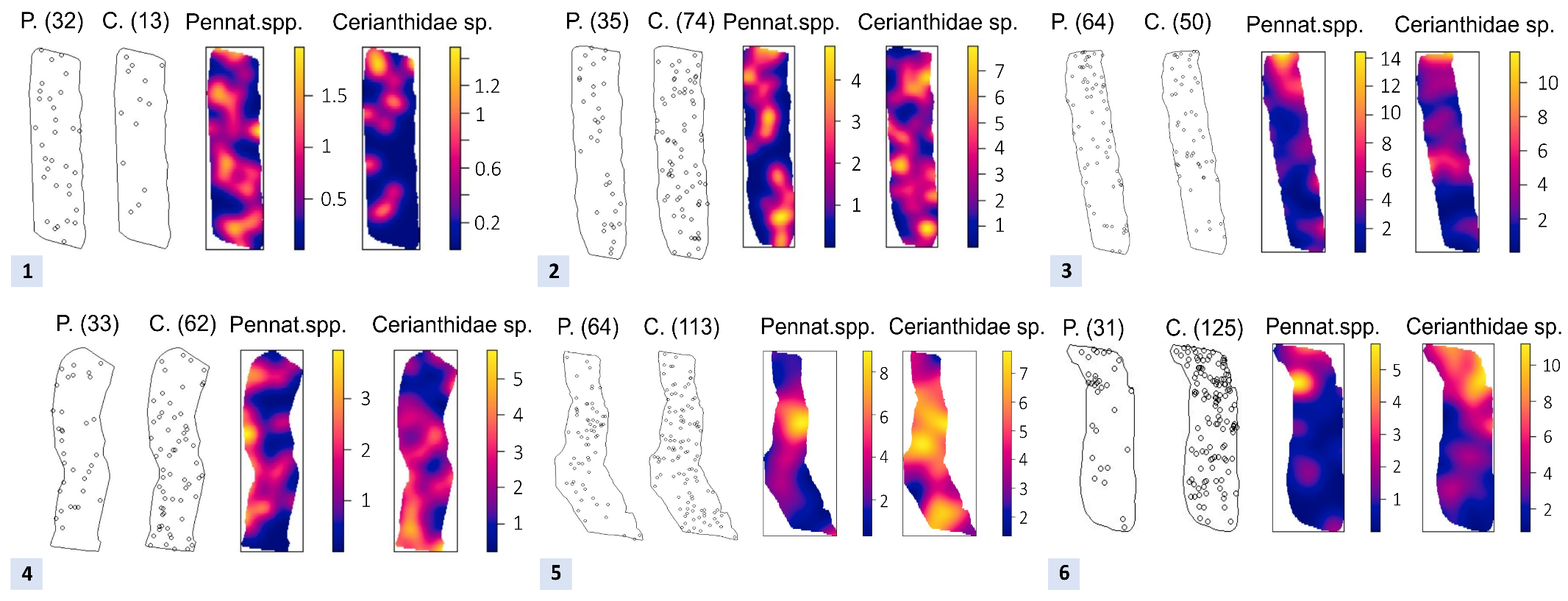


**Figure S3.** Point patterns of Pennatuloidea spp. and Cerianthidae sp. in reconstructed transects 1–6 with their respective heterogeneous Poisson models (kernel size = 1). Total Pennatuloidea spp. and Cerianthidae sp. counts and transect identifying numbers are reported for each transect. Note the varying colour scale amongst the different plots. Figure continuing on the next page.


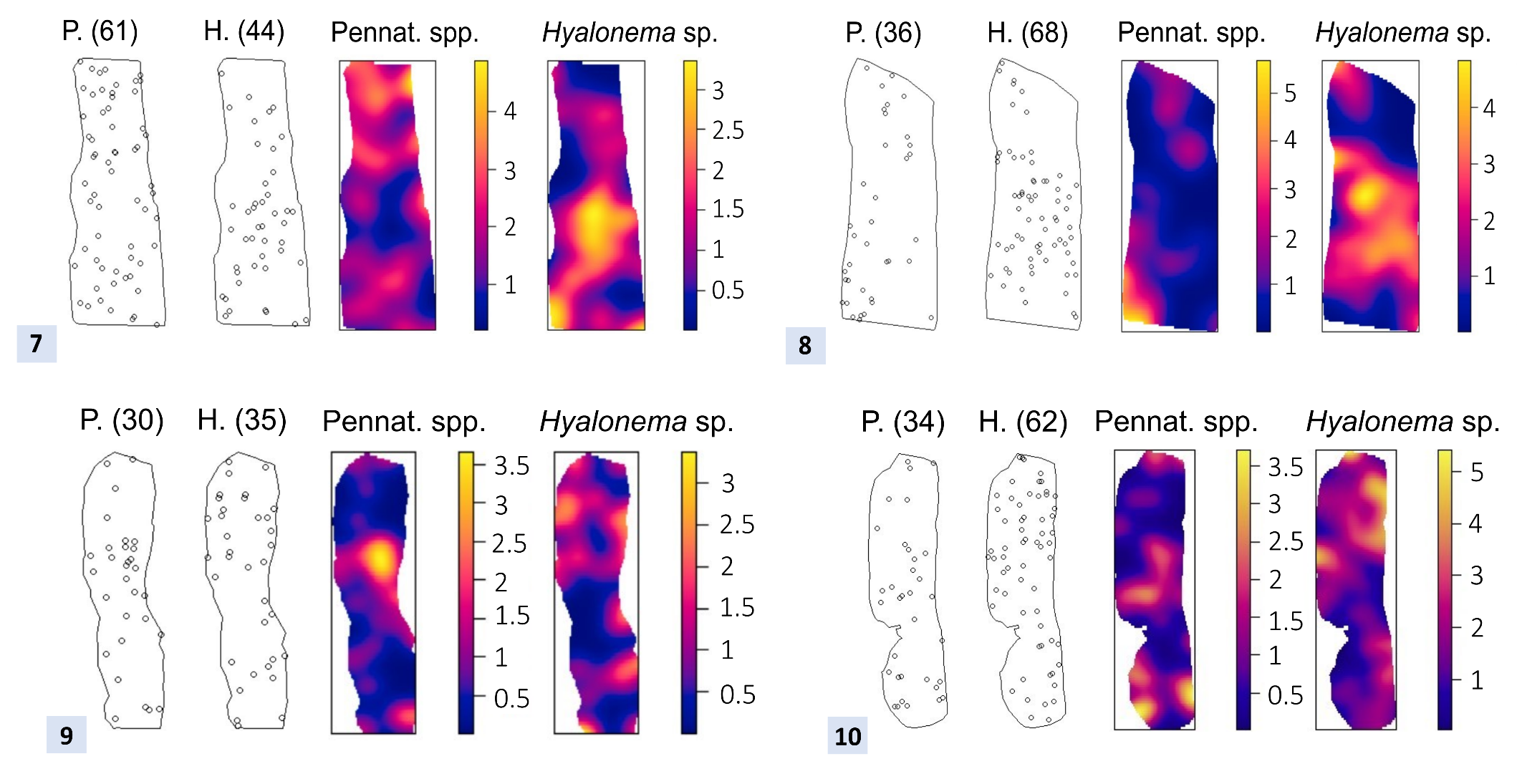


**Figure S4.** Figure S5 continued. Point patterns of Pennatuloidea spp. and *Hyalonema* sp. in reconstructed transects 7–10 with their respective heterogeneous Poisson models (kernel size = 1). Note the varying colour scale amongst the different plots.


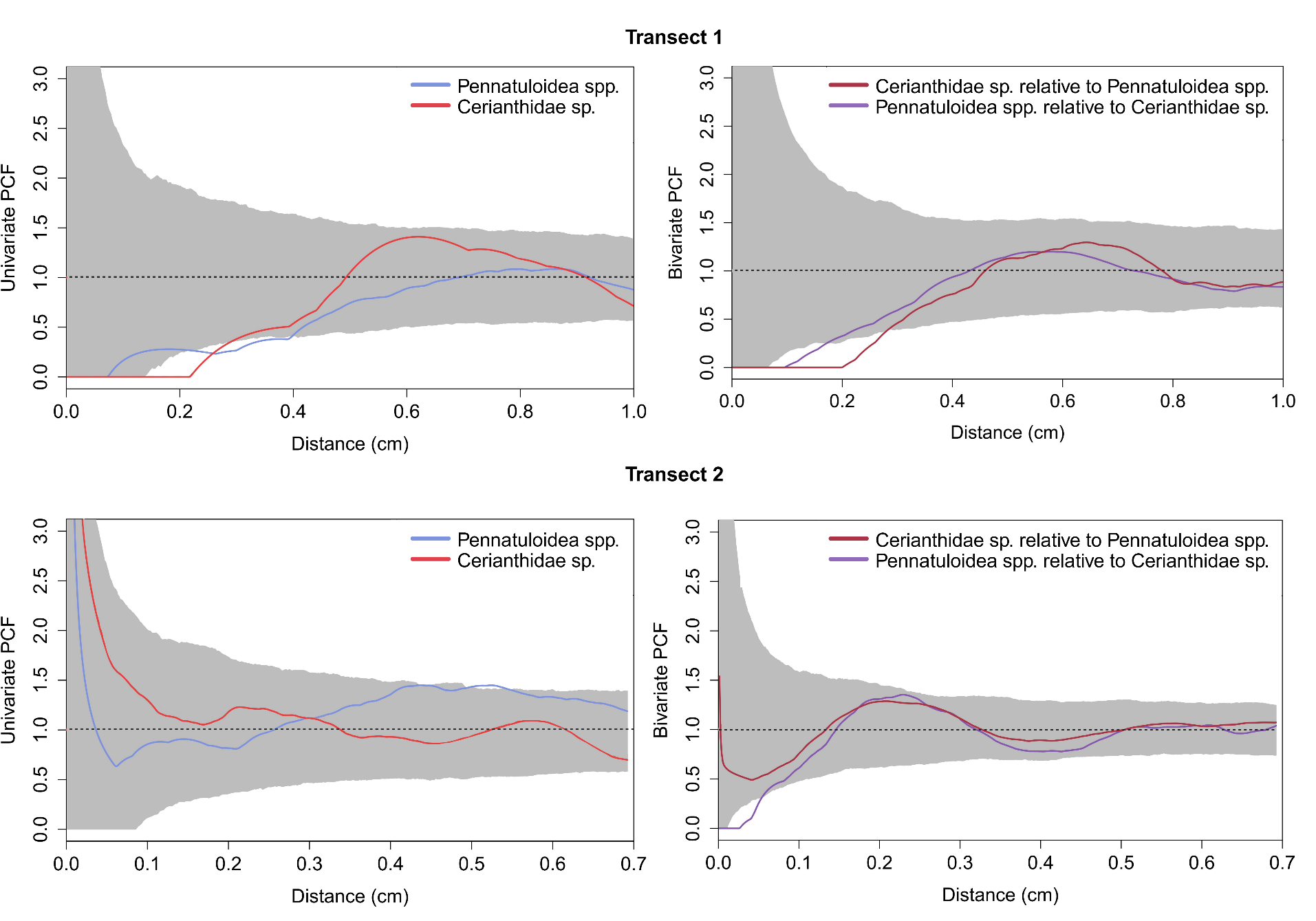


**Figure S5.** Univariate (left) and bivariate (right) PCF plots of Pennatuloidea spp. and Cerianthidae sp. in transects 1 (671 ± 3.5 m, SD) and 2 (725 ± 3.5 m, SD).


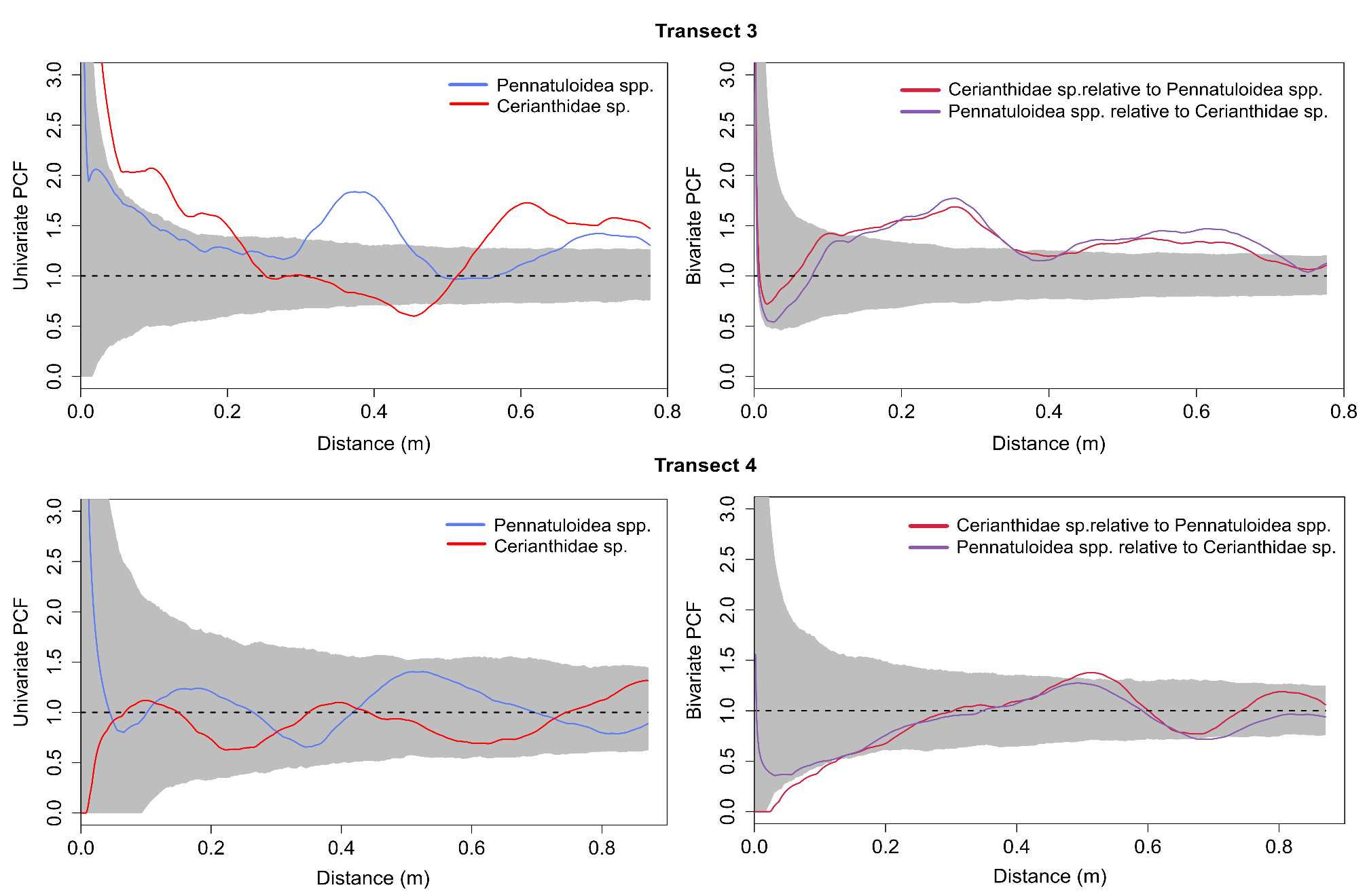


**Figure S6.** Univariate (left) and bivariate (right) PCF plots of Pennatuloidea spp. and Cerianthidae sp. in transects 3 (748 ± 1.4 m, SD) and 4 (765 ± 2.1 m, SD).


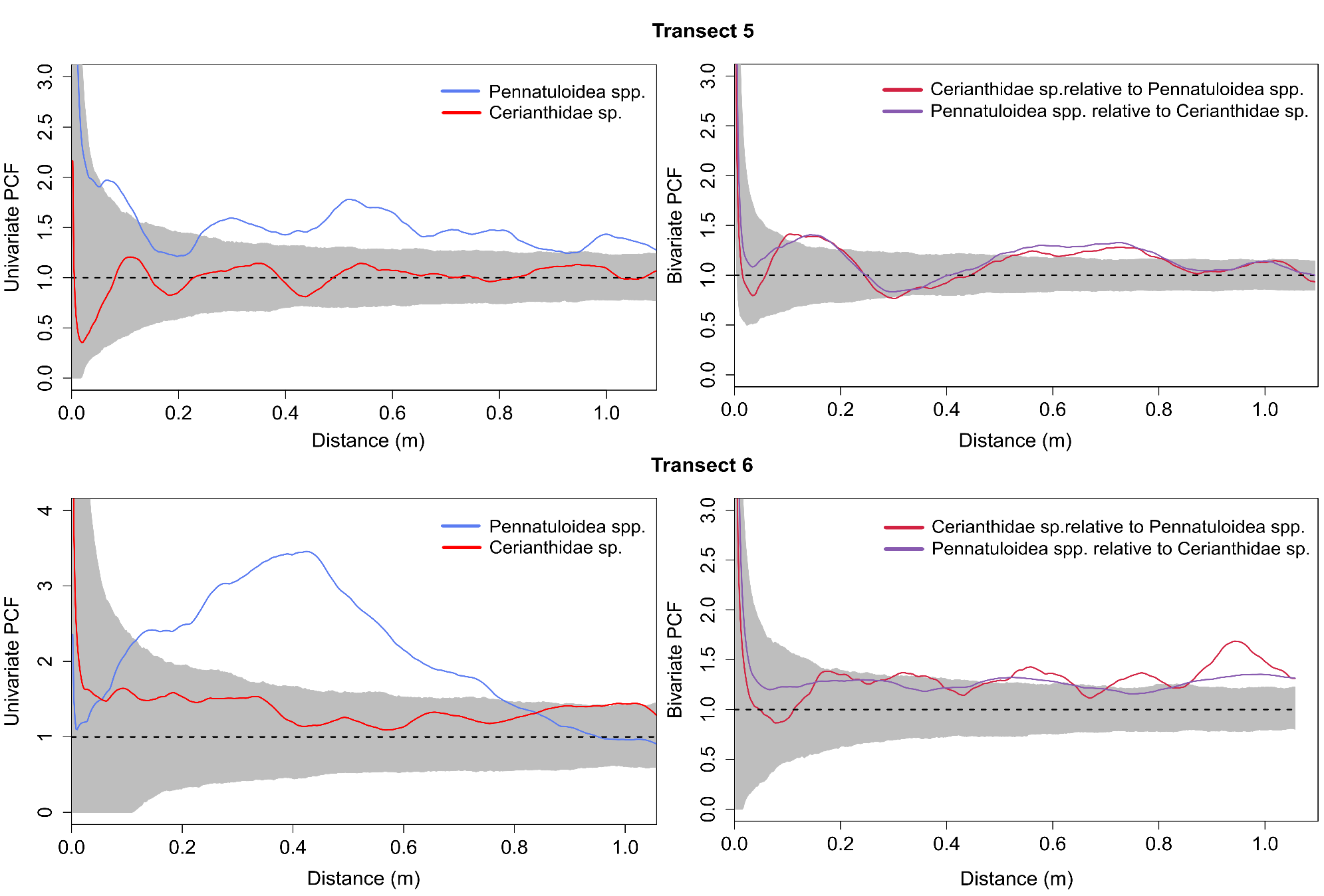


**Figure S7.** Univariate (left) and bivariate (right) PCF plots of Pennatuloidea spp. and Cerianthidae sp. in transects 5 (794 ± 2.8 m, SD) and 6 (799 ± 3.5 m, SD). Note the varying *y*-axis.


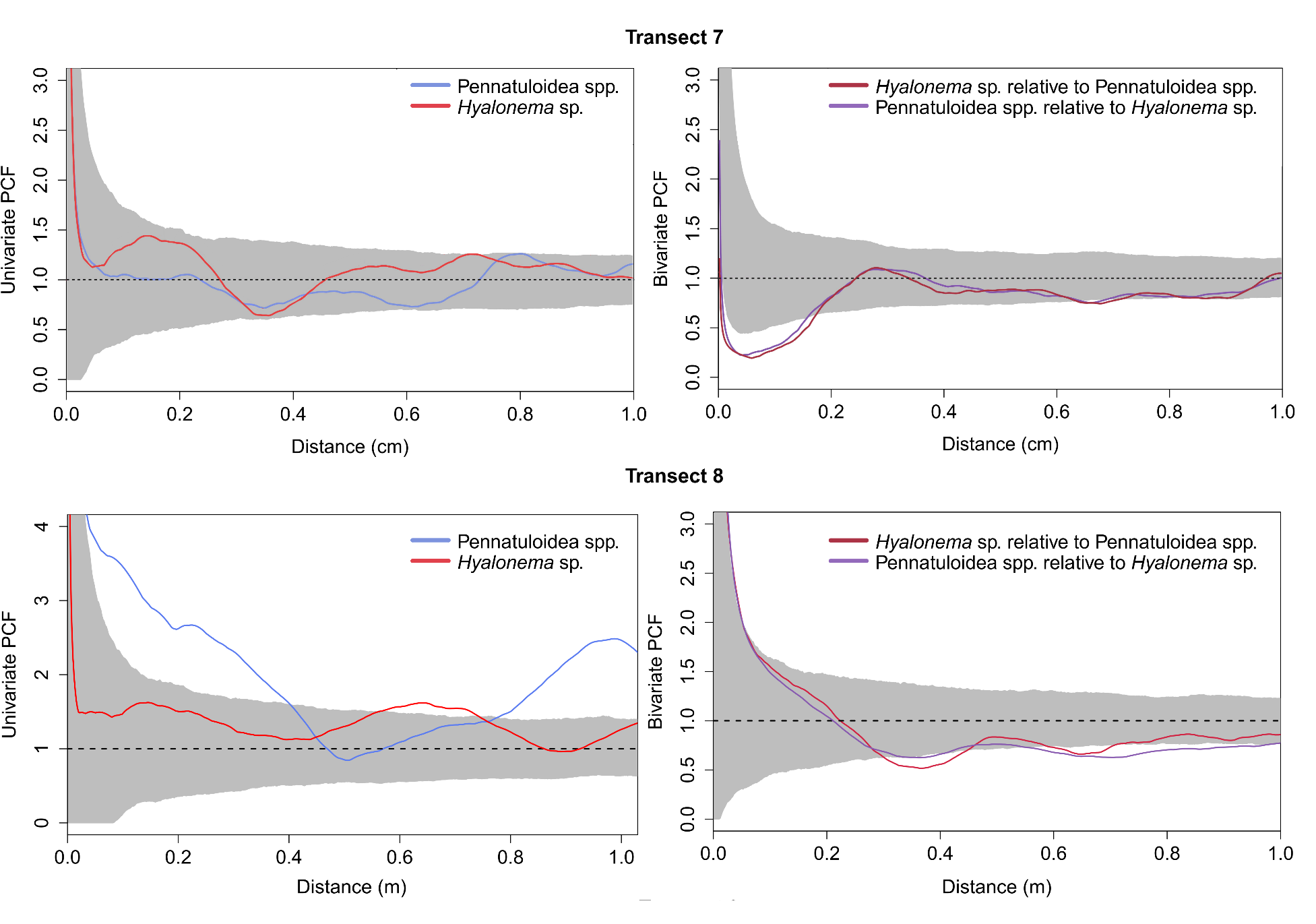


**Figure S8.** Univariate (left) and bivariate (right) PCF plots of Pennatuloidea spp. and *Hyalonema* sp. in transects 7 (981 ± 1.4 m, SD) and 8 (984 ± 2.8 m, SD). Note the varying *y*-axis.


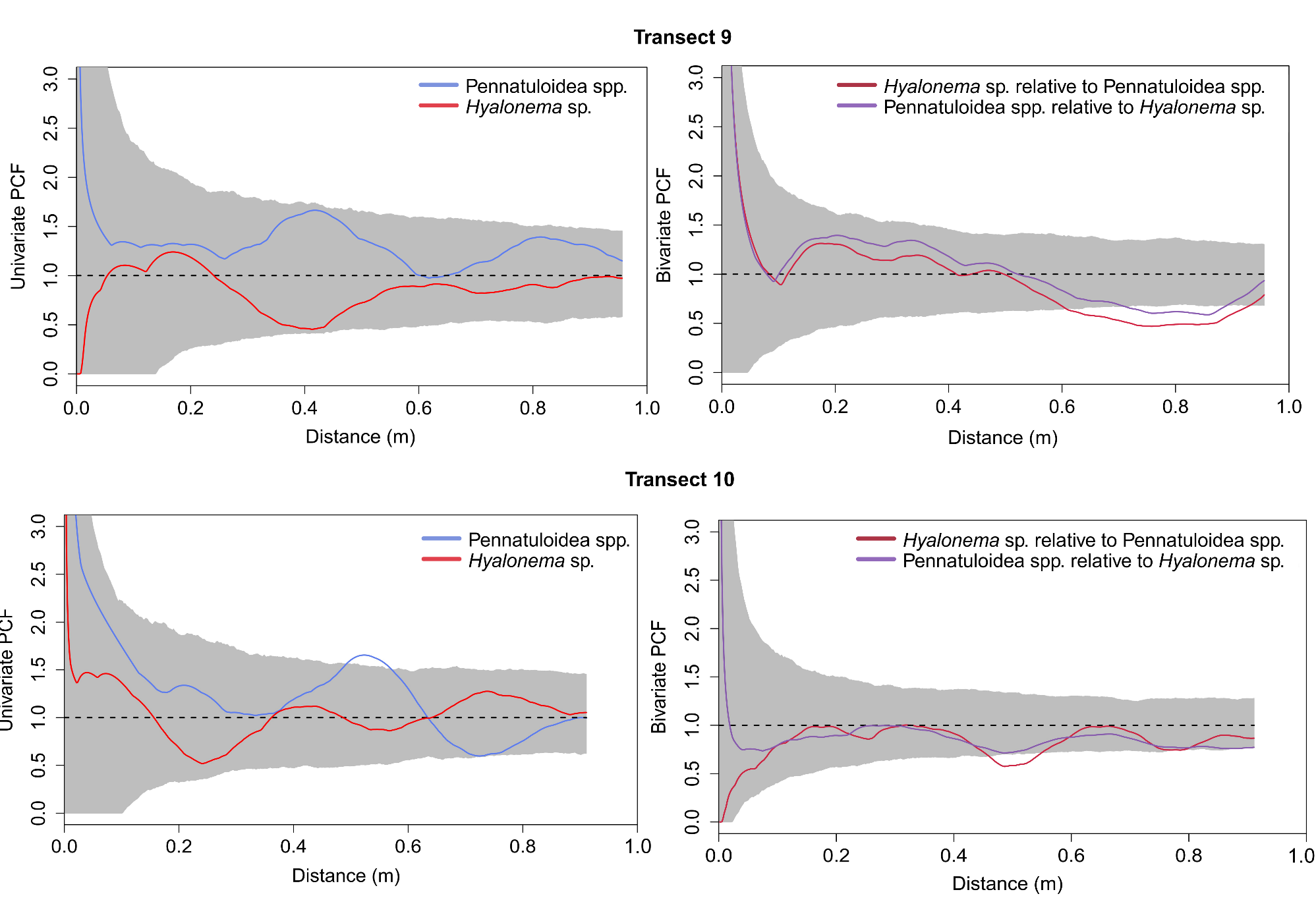


**Figure S9.** Univariate (left) and bivariate (right) PCF plots of Pennatuloidea spp. and *Hyalonema* sp. in transects 9 (991 ± 7.1 m, SD) and 10 (1000 ± 5.7 m, SD). Note the varying *y*-axis.


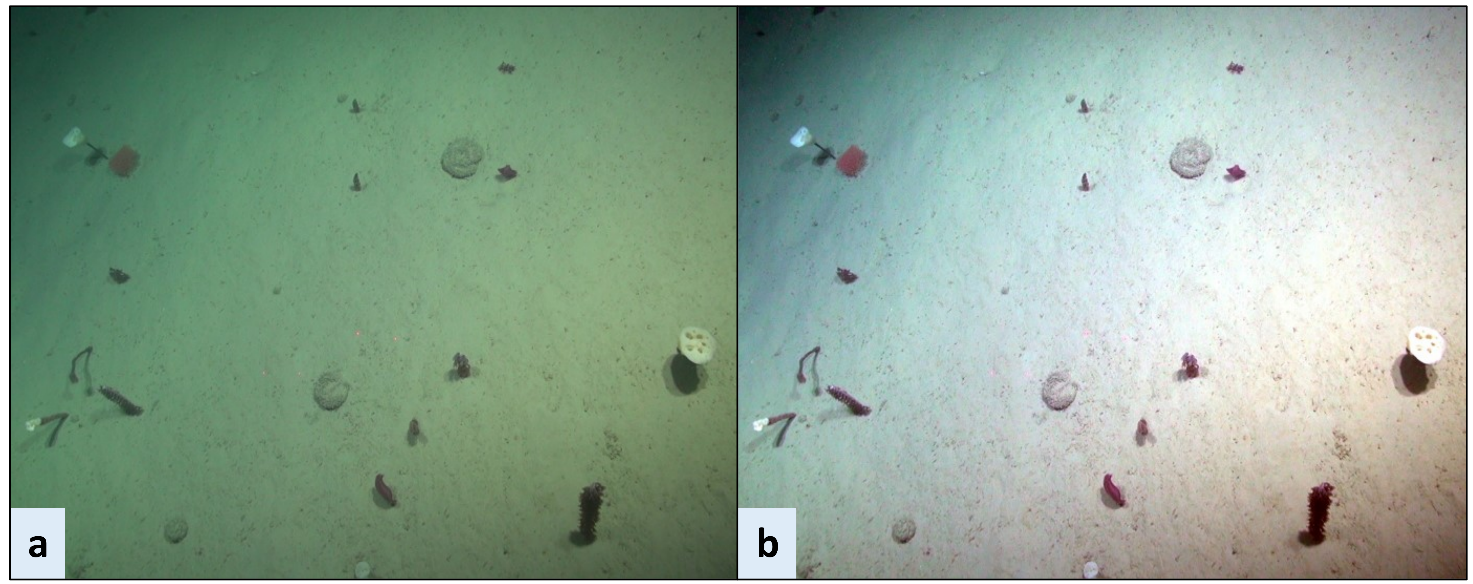


**Figure S10.** Example of frame (a) without and (b) with colour correction for underwater attenuation. To attain the correction presented in image (b), a value of 0.6 was utilised for the channel saturation percentage. Colour correction was applied using Matisse 3D v1.5.0.


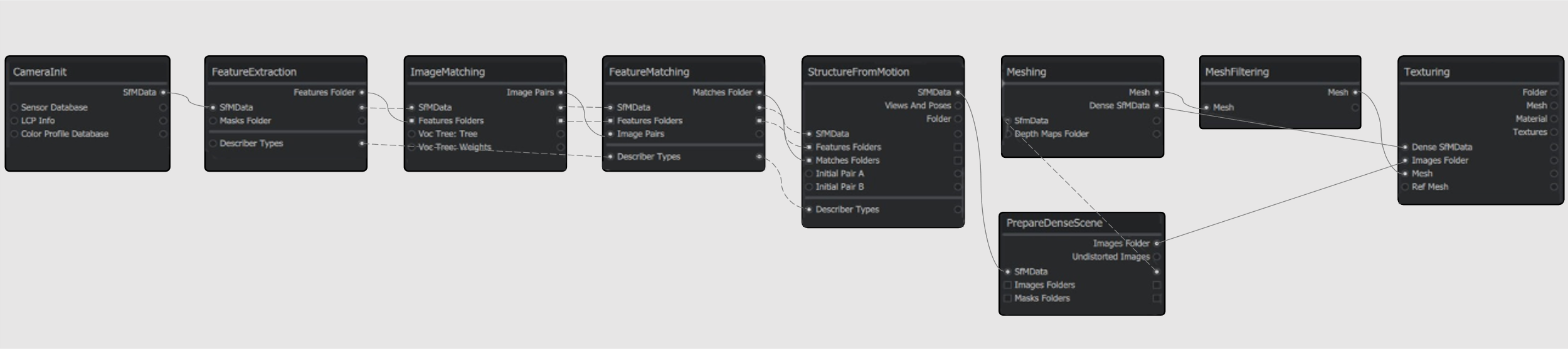


**Figure S11.** Schematic illustration of the personalised workflow developed in the 3D reconstruction software Meshroom v2023.2.0 for the purpose of the present study. This workflow was set up due to failure of depth map computation.


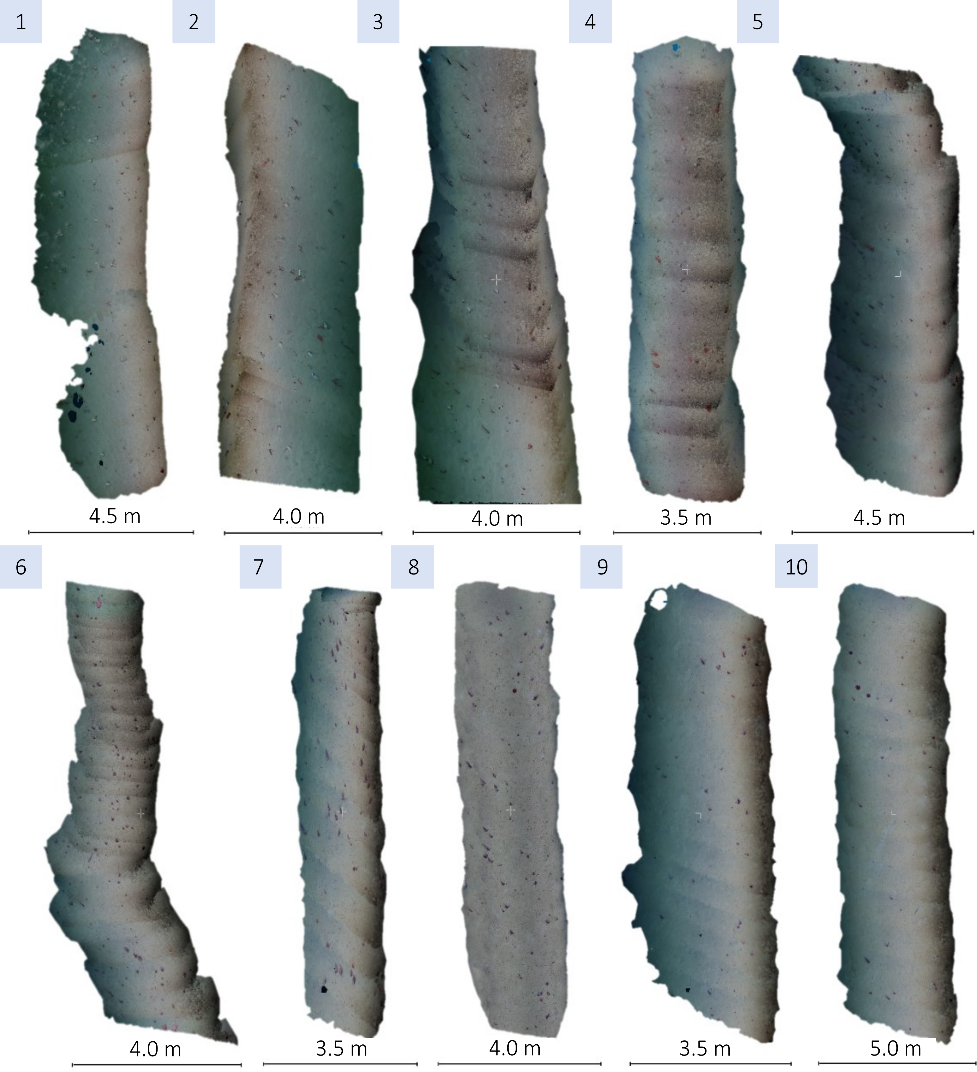


**Figure S12**. Top-down view of the 10 three-dimensional reconstructions of video transects produced in this study. A scale bar is reported for each reconstruction alongside transect identifying numbers. Three-dimensional reconstructions were generated in Meshroom v2023.2.0.


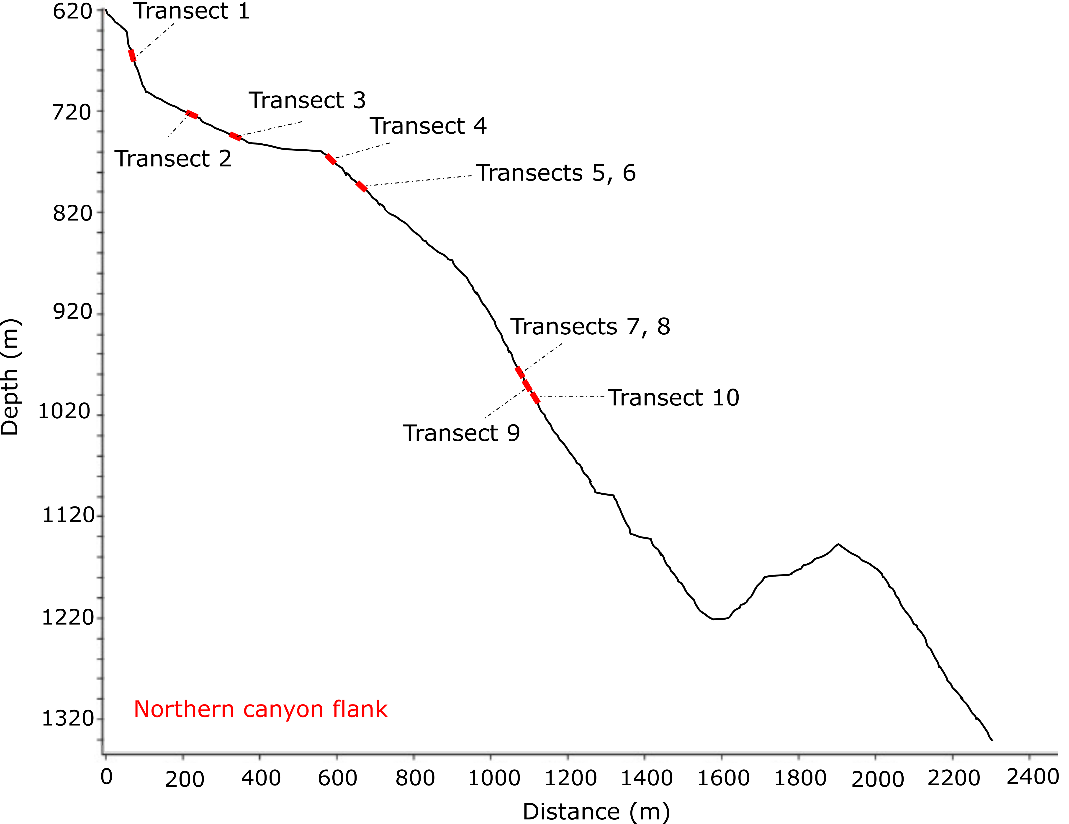


**Figure S13.** Profile of canyon flank investigated throughout this study extracted from the MESH bathymetry layer. Red segments indicate the location of each three-dimensional reconstruction.

## Univariate spatial point pattern analysis

With respect to spatial analyses, a selection of methods was implemented to describe and quantify the spatial point patterns of (i) sea pens relative to each other (univariate) and (ii) sea pens relative to a dominant non-sea-pen taxon (bivariate). For description and quantification of univariate point patterns, secondary statistics were computed first to describe the spatial distribution of sea pens, including mean nearest neighbour distance and minimum nearest neighbour distance. Secondly, heterogeneous Poisson models (i.e., density plots of the organisms’ positions) were generated using a fixed-bandwidth Kernel estimate of the intensity function^1^. Lastly, the Pair Correlation Function (PCF) was utilised as summary statistic of the point patterns. The PCF *(g(r))* is the result of a first-order derivative of the Ripley’s K function, *K(r)*, a descriptor quantifying the abundance of points expected to occur within a given distance *r* divided by the intensity of the point process, λ^2^:

$$K\left( r \right)= \frac{1}{\lambda}E (1)$$

where *K(r)* describes how the point pattern’s density changes across spatial scales, *λ* denotes the intensity of the point process, i.e., number of events per unit area, and *E* denotes the number of individuals encountered within distance *r* of a randomly selected event. In geometric terms, *K(r)* is denoted by a circle of radius *r* centred on one of the points within the point process of interest and is defined by counting the number of points that are encircled^2^ (**Figure S14a**).


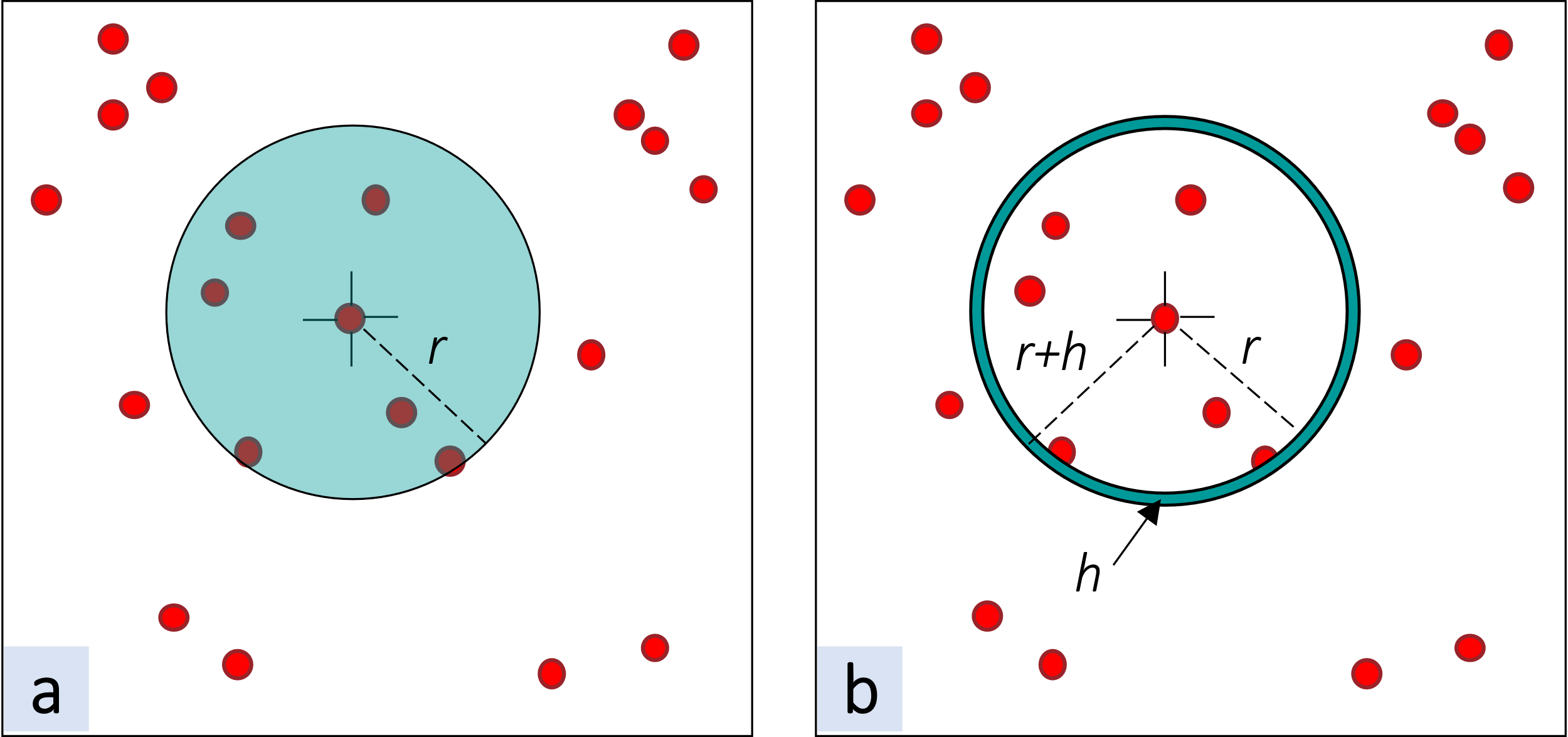


**Figure S14.** Geometric representation of Ripley’s K function (a) and the Pair Correlation Function, PCF (b). Adapted from Baddeley et al.^3^.

The PCF *(g(r))*, on the other hand, is best represented by two concentric circles of radius *r* and *r+h*, respectively—where h consists of a small increment of distance—and is defined by counting the number of points contained within the intercircle ring^2^ (**Figure S14b**). The latter captures the interpoint distance lying in the narrow ring separating *r* from *r+h*. PCF *(g(r))*, therefore, minimises the cumulative effects associated with an ever-expanding radius by relying on a two-dimensional torus shape to define a point registration field^3^. PCF analysis is commonly implemented to quantify the density of ecological objects at a growing distance *r* from a representative focal point^2,3^, thus revealing any underlying clustering or overdispersion patterns within the population of interest. The term *(g(r))* is estimated as follows:

$$g\left( r \right)= \frac{K'(r)}{2\pi r} (2)$$

where *K’(r)* denotes the first-order derivative of the Ripleys’ K function relative to distance *r*^2^. As part of the PCF, point patterns were compared with 999 Monte Carlo simulations of Complete Spatial Randomness (CSR), with statistical envelopes generated to encompass 95% of null model simulations (α = 0.05)^3^. Any excursions above or below the envelopes suggest clustering or overdispersion, respectively, the latter suggesting that the organism’s distribution is more dispersed than predicted by chance alone.

**Table S1.** Morphospecies count and density estimates for each annotated image. Species other than Pennatuloidea spp., Cerianthidae sp., and *Hyalonema* sp. were collectively classed as “other fauna” due to their limited relevance to the analyses performed in the present study. The category “other fauna” includes, in taxonomic order: *Acanella arbuscula*, Actiniaria spp., *Bathynectes* sp., Crustacea sp., Homolidae sp., Comotulida sp., *Nymphaster* sp., *Pentametrocrinus atlanticus*, *Phormosoma placenta*, Holothuridae sp., *Lepidion eques*, and *Trachyscorpia cristulata*. Pennat. = Pennatuloidea spp.; Cer. = Cerianthidae sp.; *Hyal*. = *Hyalonema* sp.

| **Image file** | **Depth (m)** | **Depth bin** | **Area (m^2^)** | **Time** | **Pennat. spp. counts** | **Pennat. spp. density (individuals m^-2^)** | **Cer. sp. counts** | **Cer. sp. density (individuals m^-2^)** | ***Hyal*. sp. counts** | ***Hyal*. sp. density (individuals m^-2^)** | **Other fauna counts** | **Other fauna density (individuals m^-2^)** |
| --- | --- | --- | --- | --- | --- | --- | --- | --- | --- | --- | --- | --- |
| DSC00291.JPG | 1328.30 | 13 | 1.05 | 00:38:30 | 0 | 0 | 1 | 0.9 | 0 | 0 | 0 | 0 |
| DSC00293.JPG | 1327.50 | 13 | 1.6 | 00:39:39 | 0 | 0 | 0 | 0 | 0 | 0 | 0 | 0 |
| DSC00300.JPG | 1327.45 | 13 | 1.16 | 00:43:00 | 0 | 0 | 0 | 0 | 0 | 0 | 0 | 0 |
| DSC00304.JPG | 1327.53 | 13 | 1.7 | 00:45:00 | 0 | 0 | 3 | 1.8 | 0 | 0 | 0 | 0 |
| DSC00317.JPG | 1317.37 | 13 | 1.4 | 00:51:30 | 0 | 0 | 0 | 0 | 0 | 0 | 0 | 0 |
| DSC00322.JPG | 1305.29 | 13 | 1.47 | 00:54:00 | 0 | 0 | 2 | 1.4 | 0 | 0 | 0 | 0 |
| DSC00331.JPG | 1297.01 | 13 | 1.14 | 00:58:30 | 0 | 0 | 0 | 0 | 0 | 0 | 0 | 0 |
| DSC00335.JPG | 1297.48 | 13 | 1.76 | 01:00:30 | 0 | 0 | 0 | 0 | 0 | 0 | 0 | 0 |
| DSC00368.JPG | 1297.13 | 13 | 0.85 | 01:17:00 | 0 | 0 | 1 | 1.2 | 0 | 0 | 0 | 0 |
| DSC00375.JPG | 1297.46 | 13 | 0.81 | 01:20:30 | 0 | 0 | 1 | 1.2 | 0 | 0 | 0 | 0 |
| DSC00386.JPG | 1294.51 | 13 | 1.67 | 01:26:00 | 0 | 0 | 1 | 0.6 | 0 | 0 | 0 | 0 |
| DSC00393.JPG | 1292.43 | 13 | 2.27 | 01:29:30 | 0 | 0 | 1 | 0.4 | 0 | 0 | 0 | 0 |
| DSC00398.JPG | 1281.51 | 13 | 1.75 | 01:32:00 | 0 | 0 | 0 | 0 | 0 | 0 | 0 | 0 |
| DSC00410.JPG | 1250.77 | 12 | 1.37 | 01:38:00 | 0 | 0 | 2 | 1.5 | 0 | 0 | 1 | 0.7 |
| DSC00441.JPG | 1209.48 | 12 | 1.05 | 01:53:30 | 0 | 0 | 1 | 1.0 | 0 | 0 | 0 | 0 |
| DSC00456.JPG | 1184.01 | 12 | 1.56 | 02:01:00 | 0 | 0 | 5 | 3.2 | 0 | 0 | 0 | 0 |
| DSC00457.JPG | 1184.01 | 12 | 0.85 | 02:01:30 | 0 | 0 | 4 | 4.7 | 0 | 0 | 0 | 0 |
| DSC00544.JPG | 1162.9 | 12 | 5.4 | 02:45:00 | 0 | 0 | 1 | 0.2 | 0 | 0 | 1 | 0.2 |
| DSC00573.JPG | 1153.58 | 11 | 4.2 | 02:59:30 | 0 | 0 | 1 | 0.2 | 0 | 0 | 0 | 0 |
| DSC00620.JPG | 1201.65 | 11 | 7.2 | 03:23:00 | 0 | 0 | 1 | 0.1 | 0 | 0 | 0 | 0 |
| **Image file** | **Depth (m)** | **Depth bin** | **Area (m^2^)** | **Time** | **Pennat. spp. counts** | **Pennat. spp. density (individuals m^-2^)** | **Cer. sp. counts** | **Cer. sp. density (individuals m^-2^)** | ***Hyal*. sp. counts** | ***Hyal*. sp. density (individuals m^-2^)** | **Other fauna counts** | **Other fauna density (individuals m^-2^)** |
| DSC00621.JPG | 1196.34 | 11 | 2.64 | 03:23:30 | 0 | 0 | 2 | 0.8 | 0 | 0 | 0 | 0 |
| DSC00626.JPG | 1188.11 | 11 | 6.85 | 03:26:00 | 0 | 0 | 0 | 0 | 0 | 0 | 0 | 0 |
| DSC00627.JPG | 1183.56 | 11 | 5.6 | 03:26:30 | 0 | 0 | 2 | 0.4 | 0 | 0 | 0 | 0 |
| DSC00629.JPG | 1179.97 | 11 | 3.78 | 03:27:30 | 0 | 0 | 2 | 0.5 | 0 | 0 | 0 | 0 |
| DSC00633.JPG | 1171.07 | 10 | 4.24 | 03:29:30 | 0 | 0 | 0 | 0 | 0 | 0 | 5 | 1.2 |
| DSC00638.JPG | 1155.87 | 10 | 2.25 | 03:39:00 | 0 | 0 | 0 | 0 | 0 | 0 | 0 | 0 |
| DSC00642.JPG | 1161.90 | 10 | 3.39 | 03:34:01 | 1 | 0 | 0 | 0 | 0 | 0 | 3 | 0.9 |
| DSC00654.JPG | 1155.35 | 10 | 3.12 | 03:40:00 | 0 | 0 | 0 | 0 | 0 | 0 | 21 | 6.7 |
| DSC00667.JPG | 1155.51 | 10 | 5.85 | 03:46:30 | 0 | 0 | 0 | 0 | 0 | 0 | 14 | 2.4 |
| DSC00669.JPG | 1155.49 | 10 | 10.95 | 03:47:31 | 0 | 0 | 1 | 0.1 | 0 | 0 | 4 | 0.4 |
| DSC00675.JPG | 1155.74 | 10 | 1.58 | 03:50:30 | 0 | 0 | 0 | 0 | 0 | 0 | 3 | 1.9 |
| DSC00678.JPG | 1156.03 | 10 | 1.36 | 03:52:00 | 0 | 0 | 0 | 0 | 0 | 0 | 7 | 5.2 |
| DSC00684.JPG | 1156.08 | 10 | 1.02 | 03:55:00 | 0 | 0 | 0 | 0 | 0 | 0 | 3 | 3.0 |
| DSC00721.JPG | 1148.79 | 10 | 6.28 | 04:13:30 | 0 | 0 | 3 | 0.5 | 0 | 0 | 5 | 0.8 |
| DSC00725.JPG | 1133.26 | 10 | 7.83 | 04:15:30 | 3 | 0.4 | 0 | 0 | 0 | 0 | 6 | 0.8 |
| DSC00726.JPG | 1129.74 | 10 | 7.47 | 04:16:00 | 0 | 0 | 0 | 0 | 0 | 0 | 2 | 0.3 |
| DSC00727.JPG | 1126.87 | 9 | 7.18 | 04:16:30 | 0 | 0 | 0 | 0 | 0 | 0 | 0 | 0 |
| DSC00728.JPG | 1122.88 | 9 | 8.46 | 04:17:00 | 2 | 0.2 | 0 | 0 | 0 | 0 | 2 | 0.2 |
| DSC00735.JPG | 1112.95 | 9 | 10.12 | 04:20:30 | 0 | 0 | 0 | 0 | 0 | 0 | 5 | 0.5 |
| DSC00737.JPG | 1113.02 | 9 | 9.06 | 04:21:30 | 1 | 0.1 | 0 | 0 | 0 | 0 | 6 | 0.7 |
| DSC00739.JPG | 1110.78 | 9 | 8.4 | 04:22:30 | 2 | 0.2 | 0 | 0 | 0 | 0 | 8 | 1.0 |
| **Image file** | **Depth (m)** | **Depth bin** | **Area (m^2^)** | **Time** | **Pennat. spp. counts** | **Pennat. spp. density (individuals m^-2^)** | **Cer. sp. counts** | **Cer. sp. density (individuals m^-2^)** | ***Hyal*. sp. counts** | ***Hyal*. sp. density (individuals m^-2^)** | **Other fauna counts** | **Other fauna density (individuals m^-2^)** |
| DSC00742.JPG | 1106.34 | 9 | 2.64 | 04:24:00 | 0 | 0 | 0 | 0 | 0 | 0 | 2 | 0.8 |
| DSC00764.JPG | 1064.07 | 8 | 8.61 | 04:35:00 | 9 | 1.0 | 0 | 0 | 0 | 0 | 3 | 0.3 |
| DSC00765.JPG | 1063.23 | 8 | 6.15 | 04:35:30 | 2 | 0.3 | 1 | 0.2 | 0 | 0 | 3 | 0.5 |
| DSC00769.JPG | 1056.96 | 8 | 3.55 | 04:37:30 | 1 | 0.3 | 5 | 1.4 | 0 | 0 | 4 | 1.1 |
| DSC00778.JPG | 1042.83 | 8 | 2.27 | 04:42:00 | 2 | 0.9 | 2 | 0.9 | 0 | 0 | 2 | 0.9 |
| DSC00784.JPG | 1033.67 | 8 | 2.8 | 04:45:00 | 4 | 1.4 | 0 | 0 | 2 | 0.7 | 0 | 0 |
| DSC00788.JPG | 1029.45 | 8 | 7.34 | 04:47:00 | 4 | 0.5 | 5 | 0.7 | 4 | 0.6 | 0 | 0 |
| DSC00800.JPG | 1019.87 | 7 | 9.76 | 04:53:00 | 1 | 0.1 | 3 | 0.3 | 8 | 0.8 | 8 | 0.8 |
| DSC00810.JPG | 1004.72 | 7 | 4.47 | 04:58:00 | 8 | 1.8 | 0 | 0 | 2 | 0.5 | 5 | 1.1 |
| DSC00814.JPG | 994.78 | 7 | 7.38 | 05:00:00 | 9 | 1.2 | 2 | 0.3 | 16 | 2.2 | 4 | 0.5 |
| DSC00820.JPG | 993.83 | 7 | 5.29 | 05:03:00 | 8 | 1.5 | 4 | 0.8 | 17 | 3.2 | 26 | 4.9 |
| DSC00825.JPG | 989.96 | 7 | 3.49 | 05:05:30 | 9 | 2.6 | 1 | 0.3 | 2 | 0.6 | 10 | 2.9 |
| DSC00829.JPG | 979.99 | 7 | 5.18 | 05:07:30 | 3 | 0.6 | 0 | 0 | 2 | 0.4 | 7 | 1.4 |
| DSC00834.JPG | 968.59 | 6 | 2.55 | 05:10:00 | 1 | 0.4 | 0 | 0 | 0 | 0 | 0 | 0 |
| DSC00836.JPG | 964.03 | 6 | 2.56 | 05:11:00 | 0 | 0 | 2 | 0.8 | 0 | 0 | 0 | 0 |
| DSC00840.JPG | 955.7 | 6 | 3.54 | 05:13:00 | 3 | 0.8 | 1 | 0.3 | 0 | 0 | 1 | 0.3 |
| DSC00848.JPG | 949.44 | 6 | 2.54 | 05:17:00 | 1 | 0.4 | 0 | 0 | 0 | 0 | 3 | 1.2 |
| DSC00849.JPG | 947.68 | 6 | 2.59 | 05:17:30 | 1 | 0.4 | 7 | 2.7 | 0 | 0 | 6 | 2.3 |
| DSC00854.JPG | 937.51 | 6 | 3.5 | 05:20:00 | 4 | 1.1 | 0 | 0 | 0 | 0 | 3 | 0.9 |
| DSC00860.JPG | 926.69 | 5 | 3.93 | 05:23:00 | 2 | 0.5 | 3 | 0.8 | 0 | 0 | 3 | 0.8 |
| DSC00865.JPG | 918.36 | 5 | 4.19 | 05:25:30 | 0 | 0 | 10 | 2.4 | 0 | 0 | 0 | 0 |
| **Image file** | **Depth (m)** | **Depth bin** | **Area (m^2^)** | **Time** | **Pennat. spp. counts** | **Pennat. spp. density (individuals m^-2^)** | **Cer. sp. counts** | **Cer. sp. density (individuals m^-2^)** | ***Hyal*. sp. counts** | ***Hyal*. sp. density (individuals m^-2^)** | **Other fauna counts** | **Other fauna density (individuals m^-2^)** |
| DSC00875.JPG | 905.28 | 5 | 2.64 | 05:30:30 | 0 | 0 | 5 | 1.9 | 0 | 0 | 1 | 0.4 |
| DSC00888.JPG | 890.96 | 5 | 4.17 | 05:37:00 | 8 | 1.9 | 18 | 4.3 | 5 | 1.2 | 52 | 12.5 |
| DSC00891.JPG | 886.07 | 5 | 3.22 | 05:38:30 | 7 | 2.2 | 15 | 4.7 | 14 | 4.4 | 29 | 9.0 |
| DSC00914.JPG | 857.58 | 5 | 2.52 | 05:50:00 | 4 | 1.6 | 9 | 3.6 | 13 | 5.2 | 8 | 3.2 |
| DSC00920.JPG | 847.48 | 4 | 3.38 | 05:53:01 | 4 | 1.2 | 3 | 0.9 | 0 | 0 | 4 | 1.2 |
| DSC00922.JPG | 843.02 | 4 | 4.31 | 05:54:00 | 1 | 0.2 | 1 | 0.2 | 0 | 0 | 0 | 0 |
| DSC00929.JPG | 839.89 | 4 | 1.88 | 05:57:30 | 1 | 0.5 | 8 | 4.3 | 0 | 0 | 3 | 1.6 |
| DSC00936.JPG | 832.9 | 4 | 1.23 | 06:01:00 | 0 | 0 | 34 | 27.6 | 0 | 0 | 4 | 3.3 |
| DSC00937.JPG | 832.30 | 4 | 1.82 | 06:01:30 | 0 | 0 | 24 | 13.2 | 0 | 0 | 1 | 0.6 |
| DSC00948.JPG | 825.88 | 3 | 2 | 06:07:00 | 0 | 0 | 1 | 0.5 | 0 | 0 | 1 | 0.5 |
| DSC00965.JPG | 806.87 | 3 | 1.76 | 06:15:30 | 1 | 0.6 | 8 | 4.5 | 0 | 0 | 0 | 0 |
| DSC00983.JPG | 795.43 | 3 | 4.53 | 06:24:30 | 23 | 5.1 | 23 | 5.1 | 0 | 0 | 1 | 0.2 |
| DSC00985.JPG | 794.83 | 3 | 2.89 | 06:25:30 | 7 | 2.4 | 17 | 5.9 | 0 | 0 | 1 | 0.3 |
| DSC00997.JPG | 785.73 | 3 | 4.06 | 06:31:30 | 7 | 1.7 | 26 | 6.4 | 0 | 0 | 2 | 0.5 |
| DSC01002.JPG | 781.14 | 3 | 3.01 | 06:34:00 | 7 | 2.3 | 8 | 2.7 | 0 | 0 | 2 | 0.7 |
| DSC01011.JPG | 771.78 | 2 | 4.31 | 06:38:30 | 5 | 1.2 | 18 | 4.2 | 0 | 0 | 3 | 0.7 |
| DSC01012.JPG | 770.58 | 2 | 3.45 | 06:39:00 | 6 | 1.7 | 17 | 4.9 | 0 | 0 | 1 | 0.3 |
| DSC01013.JPG | 769.54 | 2 | 3.37 | 06:39:30 | 2 | 0.6 | 18 | 5.3 | 0 | 0 | 2 | 0.6 |
| DSC01018.JPG | 763.94 | 2 | 3.75 | 06:42:00 | 8 | 2.1 | 23 | 6.1 | 0 | 0 | 0 | 0 |
| DSC01023.JPG | 758.65 | 2 | 5.38 | 06:44:30 | 16 | 3.0 | 9 | 1.7 | 0 | 0 | 0 | 0 |
| DSC01037.JPG | 748.07 | 2 | 6.01 | 06:51:30 | 20 | 3.3 | 24 | 4.0 | 0 | 0 | 13 | 2.2 |
| **Image file** | **Depth (m)** | **Depth bin** | **Area (m^2^)** | **Time** | **Pennat. spp. counts** | **Pennat. spp. density (individuals m^-2^)** | **Cer. sp. counts** | **Cer. sp. density (individuals m^-2^)** | ***Hyal*. sp. counts** | ***Hyal*. sp. density (individuals m^-2^)** | **Other fauna counts** | **Other fauna density (individuals m^-2^)** |
| DSC01073.JPG | 720.88 | 1 | 5.49 | 07:09:30 | 15 | 2.7 | 14 | 2.6 | 0 | 0 | 12 | 2.2 |
| DSC01222.JPG | 677.54 | 1 | 5.45 | 08:24:00 | 11 | 2.0 | 8 | 1.5 | 0 | 0 | 5 | 0.9 |
| DSC01246.JPG | 682.03 | 1 | 8.49 | 08:36:00 | 0 | 0 | 14 | 1.6 | 0 | 0 | 9 | 1.1 |
| DSC01247.JPG | 682.94 | 1 | 6.26 | 08:36:30 | 0 | 0 | 16 | 2.6 | 0 | 0 | 1 | 0.2 |
| DSC01248.JPG | 682.90 | 1 | 7.65 | 08:37:00 | 0 | 0 | 10 | 1.3 | 0 | 0 | 5 | 0.7 |
| DSC01249.JPG | 684.44 | 1 | 4.78 | 08:37:30 | 0 | 0 | 13 | 2.7 | 0 | 0 | 3 | 0.6 |
| DSC01250.JPG | 683.84 | 1 | 5.32 | 08:38:00 | 0 | 0 | 20 | 3.8 | 0 | 0 | 11 | 2.1 |
| DSC01251.JPG | 684.10 | 1 | 5.09 | 08:38:30 | 0 | 0 | 14 | 2.8 | 0 | 0 | 7 | 1.4 |
| DSC01252.JPG | 684.58 | 1 | 5.03 | 08:39:01 | 0 | 0 | 36 | 7.2 | 0 | 0 | 2 | 0.4 |
| DSC01253.JPG | 684.46 | 1 | 3.58 | 08:39:30 | 1 | 0.3 | 10 | 2.8 | 0 | 0 | 5 | 1.4 |
| DSC01254.JPG | 684.22 | 1 | 4.7 | 08:40:00 | 0 | 0 | 11 | 2.3 | 0 | 0 | 1 | 0.2 |
| DSC01255.JPG | 683.58 | 1 | 4.53 | 08:40:30 | 4 | 0.9 | 12 | 2.6 | 0 | 0 | 5 | 1.1 |
| DSC01258.JPG | 683.23 | 1 | 4.93 | 08:41:00 | 8 | 1.6 | 8 | 1.6 | 0 | 0 | 2 | 0.4 |
| DSC01260.JPG | 683.55 | 1 | 5.93 | 08:42:00 | 9 | 1.5 | 17 | 2.9 | 0 | 0 | 1 | 0.2 |
| DSC01261.JPG | 683.50 | 1 | 5.98 | 08:42:30 | 9 | 1.5 | 12 | 2.0 | 0 | 0 | 1 | 0.2 |
| DSC01262.JPG | 683.33 | 1 | 6.06 | 08:43:00 | 9 | 1.5 | 14 | 2.3 | 0 | 0 | 1 | 0.2 |
| DSC01263.JPG | 682.81 | 1 | 5.67 | 08:43:30 | 4 | 0.7 | 7 | 1.2 | 0 | 0 | 4 | 0.7 |

**Table S2.** Mean density of Pennatuloidea spp., Cerianthidae sp., and Hyalonema sp. across depth bins.

| **Depth bin ID** | **Depth (m)** | **Area (m^2^)** | **Pennatuloidea spp. mean density (ind. m^-2^)** | **Cerianthidae sp. mean density (ind. m^-2^)** | ***Hyalonema* sp. mean density (ind. m^-2^)** |
| --- | --- | --- | --- | --- | --- |
| 1 | 686.5–736.5 | 94.9 | 0.7 | 2.4 | 0 |
| 2 | 736.51–786.51 | 26.3 | 2.0 | 4.4 | 0 |
| 3 | 786.52–836.52 | 18.3 | 2.0 | 4.2 | 0 |
| 4 | 836.53–886.53 | 12.6 | 0.4 | 9.2 | 0 |
| 5 | 886.54–936.54 | 20.7 | 1.0 | 2.9 | 1.8 |
| 6 | 936.55–986.55 | 17.3 | 0.5 | 0.6 | 0 |
| 7 | 986.56–1036.56 | 35.6 | 1.3 | 0.3 | 1.3 |
| 8 | 1036.57–1086.57 | 30.7 | 0.8 | 0.5 | 0.2 |
| 9 | 1086.58–1136.58 | 45.9 | 0.1 | 0 | 0 |
| 10 | 1136.59–1186.59 | 55.3 | 0.1 | 0 | 0 |
| 11 | 1186.6–1236.6 | 30.3 | 0 | 0.3 | 0 |
| 12 | 1236.61–1286.61 | 10.2 | 0 | 2.1 | 0 |
| 13 | 1286.62–1336.62 | 18.6 | 0 | 0.6 | 0 |

**Table S3.** Species count and density estimates for each investigated transect. Species other than Pennatuloidea spp., Cerianthidae sp., and *Hyalonema* sp. were collectively classed as “other fauna” due to their limited relevance to the analyses performed in the present study. The category “other fauna” includes, in taxonomic order: *Acanella arbuscula*, Actiniaria spp., *Bathynectes* sp., Crustacea sp., Homolidae sp., Comotulida sp., *Nymphaster* sp., *Pentametrocrinus atlanticus*, *Phormosoma placenta*, Holothuridae sp., *Lepidion eques*, and *Trachyscorpia cristulata*. Pennat. = Pennatuloidea spp.; Cer. = Cerianthidae sp.; *Hyal*. = *Hyalonema* sp.

| **Transect ID** | **Mean depth ± SD (m)** | **Transect area (m^2^)** | **Pennat. spp. counts** | **Pennat. spp. density (individuals/m^2^)** | **Cer. sp. counts** | **Cer. sp. density (individuals/m^2^)** | ***Hyal*. sp. counts** | ***Hyal*. sp. density (individuals/m^2^)** | **Other fauna counts** | **Other fauna density (individuals/m^2^)** |
| --- | --- | --- | --- | --- | --- | --- | --- | --- | --- | --- |
| 1 | 671 ± 3.5 | 52.5 | 32 | 0.6 | 13 | 0.3 | 0 | 0 | 10 | 0.2 |
| 2 | 725 ± 3.5 | 28.3 | 35 | 1.2 | 74 | 2.6 | 0 | 0 | 11 | 0.4 |
| 3 | 748 ± 1.4 | 20.8 | 64 | 3.1 | 50 | 2.4 | 0 | 0 | 5 | 0.2 |
| 4 | 765 ± 2.1 | 31.8 | 33 | 1 | 62 | 2 | 0 | 0 | 0 | 0 |
| 5 | 794 ± 2.8 | 24.4 | 64 | 2.6 | 113 | 4.6 | 0 | 0 | 11 | 0.5 |
| 6 | 799 ± 3.5 | 34.5 | 31 | 0.9 | 125 | 3.6 | 0 | 0 | 8 | 0.2 |
| 7 | 981 ± 1.4 | 39.4 | 61 | 1.6 | 11 | 0.3 | 44 | 1.1 | 7 | 0.2 |
| 8 | 984 ± 2.8 | 47.9 | 36 | 0.8 | 8 | 0.2 | 68 | 1.4 | 5 | 0.1 |
| 9 | 991 ± 7.1 | 49.4 | 30 | 0.6 | 5 | 0.1 | 35 | 0.7 | 12 | 0.2 |
| 10 | 1000 ± 5.7 | 42.7 | 34 | 0.8 | 8 | 0.2 | 62 | 1.5 | 8 | 0.2 |

**Table S4.** Summary of all the models that were fitted to the univariate point patterns showing clustering. The models were applied both to the entire function and to the clustering peak alone. ID = transect ID, CSR = Complete Spatial Randomness, HP = heterogeneous Poisson, HTC = homogeneous Thomas cluster, ITC = inhomogeneous Thomas cluster, x = relative to the x direction, y = relative to the y direction, x + y = relative to x–y directions, – = relative to 1 (~ 1), i.e., stationary process.

|  | **Univariate PCFs** | | | | | | | | | | | | | | | | | |
| --- | --- | --- | --- | --- | --- | --- | --- | --- | --- | --- | --- | --- | --- | --- | --- | --- | --- | --- |
|  | **Entire function** | | | | | | | | | **Clustering peak** | | | | | | | | |
|  | **CSR** | **HP** | | | | **HTC** | **ITC** | | | **CSR** | **HP** | | | | **HTC** | **ITC** | | |
| **ID** | — | *x* | *y* | *x* + *y* | Density | — | *x* | *y* | *x* + *y* | — | *x* | *y* | *x* + *y* | Density | — | *x* | *y* | *x* + *y* |
| 3 | 0.005 | 0.002 | 0.367 | 0.703 | **0.831** | 0.002 | 0.004 | 0.323 | 0.701 | 0.007 | 0.006 | 0323 | 0.711 | **0.844** | 0.005 | 0.005 | 0.351 | 0.760 |
| 5 | 0.001 | 0.001 | 0.002 | 0.002 | **0.997** | 0.001 | 0.001 | 0.004 | 0.001 | 0.001 | 0.001 | 0.003 | 0.001 | **0.998** | 0.001 | 0.001 | 0.002 | 0.003 |
| 6 | 0.001 | 0.002 | 0.027 | 0.057 | **0.556** | 0.001 | 0.002 | 0.027 | 0.028 | 0.001 | 0.002 | 0.031 | 0.043 | **0.549** | 0.001 | 0.001 | 0.025 | 0.039 |
| 8 | 0.003 | 0.116 | 0.011 | 0.201 | **0.342** | 0.006 | 0.117 | 0.012 | 0.207 | 0.003 | 0.109 | 0.015 | 0.219 | **0.362** | 0.01 | 0.109 | 0.01 | 0.247 |
| 10 | 0.47 | 0.422 | 0.589 | 0.583 | 0.419 | 0.47 | 0.42 | 0.574 | **0.639** | 0.47 | 0.406 | 0.585 | 0.584 | 0.458 | 0.463 | 0.386 | 0.600 | **0.622** |

|  | **SC** | | | | | | | | | **HC** |
| --- | --- | --- | --- | --- | --- | --- | --- | --- | --- | --- |
| **ID** | Kappa = 0.1 | Kappa = 0.2 | Kappa = 0.3 | Kappa = 0.4 | Kappa = 0.5 | Kappa = 0.6 | Kappa = 0.7 | Kappa = 0.8 | Kappa = 0.9 | — |
| 3 | 0.006 | 0.008 | 0.004 | 0.004 | 0.005 | 0.005 | 0.005 | 0.008 | 0.005 | 0.002 |

**Table S5.** Summary of all the models that were fitted to the univariate point patterns showing overdispersion. ID = transect ID, SC = soft-core process, HC = hard-core process, kappa (0.1 –0.9) = all possible values of the exponent kappa of the softcore() function in R.

**Table S6.** Summary of all the models that were fitted to the bivariate point patterns showing clustering. The models were applied both to the entire function and to the clustering peak alone. ID = transect ID, HP = heterogeneous Poisson, Density (P) = heterogeneous Poisson model centred on the density of Pennatuloidea spp., Density (C) = heterogeneous Poisson model centred on the density of Cerianthidae sp., Density (J) = heterogeneous Poisson model centred on the joint density of Pennatuloidea spp. and Cerianthidae sp.

|  | **Bivariate PCFs** | | | | | |
| --- | --- | --- | --- | --- | --- | --- |
|  | **Entire function** | | | **Clustering peak** | | |
|  | **HP** | | | **HP** | | |
| **ID** | Density (P) | Density (C) | Density (J) | Density (P) | Density (C) | Density (J) |
| 3 | 0.895 | **0.945** | 0.915 | 0.897 | **0.960** | 0.929 |
| 4 | **0.098** | 0.092 | 0.057 | 0.090 | **0.104** | 0.05 |
| 5 | 0.780 | 0.786 | **0.816** | 0.799 | 0.759 | **0.814** |
| 6 | 0.466 | **0.886** | 0.866 | 0.461 | **0.894** | 0.808 |

**Table S7.** Distance statistics from Pennatuloidea spp. to the nearest dominant non-Pennatuloidea taxon for each investigated transect. Mean depth is also reported for reference.

| **Transect ID** | **Min. distance from nearest Cer. (m)** | **Mean distance from nearest Cer. (m)** | **Min. distance from nearest *Hyal*. (m)** | **Mean distance from nearest *Hyal*. (m)** | **Mean depth ± SD (m)** |
| --- | --- | --- | --- | --- | --- |
| 1 | 0.4 | 1.1 | — | — | 671 ± 3.5 |
| 2 | 0.04 | 0.3 | — | — | 725 ± 3.5 |
| 3 | 0.04 | 0.3 | — | — | 748 ± 1.4 |
| 4 | 0.1 | 0.4 | — | — | 765 ± 2.1 |
| 5 | 0.0002 | 0.2 | — | — | 794 ± 2.8 |
| 6 | 0.05 | 0.2 | — | — | 799 ± 3.5 |
| 7 | — | — | 0.06 | 0.5 | 981 ± 1.4 |
| 8 | — | — | 0.01 | 0.4 | 984 ± 2.8 |
| 9 | — | — | 0.09 | 0.6 | 991 ± 7.1 |
| 10 | — | — | 0.1 | 0.4 | 1000 ± 5.7 |

**Table S8.** Metadata of reconstructed transects. The value reported for the slope refers to the dip of the bathymetry data.

| **Transect ID** | **Aligned images** | **Length (m)** | **Transect time (mm:ss)** | **ROV speed (m∙s^-1^)** | **Slope (°)** | **Number of dense cloud points** | **Mean depth ± SD (m)** |
| --- | --- | --- | --- | --- | --- | --- | --- |
| 1 | 436 | 13.6 | 01:57 | 0.12 | 55 | 116,465 | 671 ± 3.5 |
| 2 | 602 | 10.6 | 01:56 | 0.09 | 24 | 425,144 | 725 ± 3.5 |
| 3 | 570 | 9.8 | 01:54 | 0.09 | 6 | 67,067 | 748 ± 1.4 |
| 4 | 468 | 11.2 | 02:15 | 0.08 | 41 | 224,153 | 765 ± 2.1 |
| 5 | 633 | 9.9 | 03:56 | 0.04 | 41 | 347,360 | 794 ± 2.8 |
| 6 | 632 | 10.2 | 03:55 | 0.04 | 41 | 539,155 | 799 ± 3.5 |
| 7 | 406 | 10.6 | 01:04 | 0.17 | 47 | 222,673 | 981 ± 1.4 |
| 8 | 405 | 10.3 | 01:04 | 0.16 | 47 | 136,638 | 984 ± 2.8 |
| 9 | 406 | 13 | 04:04 | 0.05 | 47 | 213,887 | 991 ± 7.1 |
| 10 | 265 | 12.4 | 01:20 | 0.16 | 47 | 143,403 | 1000 ± 5.7 |
| **Total** | **4823** | **111.6** | **23:43** | **—** | **—** | **2,435,945** | **—** |

# **References**

1. Price, D. M. *et al.* Fine-Scale Heterogeneity of a Cold-Water Coral Reef and Its Influence on the Distribution of Associated Taxa. *Front. Mar. Sci.* **8**, 556313 (2021).

2. Illian, J., Penttinen, A., Stoyan, H. & Stoyan, D. *Statistical Analysis and Modelling of Spatial Point Patterns*. (John Wiley & Sons, 2008).

3. Baddeley, A., Rubak, E. & Turner, R. *Spatial Point Patterns: Methodology and Applications with R*. (CRC Press, 2015).
